# Adolescent Social Isolation Induces Persistent Impairments in Emotional Discrimination and Helping Behavior

**DOI:** 10.1523/ENEURO.0441-25.2026

**Published:** 2026-07-07

**Authors:** Hyo Gun Lee, Ju Lu, Yi Zuo

**Affiliations:** ^1^Department of Molecular, Cell and Developmental Biology, University of California Santa Cruz, Santa Cruz, California 95064; ^2^Department of Biological Sciences, Lehigh University, Bethlehem, Pennsylvania 18015

**Keywords:** adolescence, allogrooming, emotional discrimination, prosocial behavior, social isolation

## Abstract

Prosocial behaviors, helpful actions directed toward the well-being of others, are an essential component of social behavior. Adolescence is a particularly sensitive period for the development of social behaviors, yet how social experience during this window shapes prosocial behavior remains poorly understood. Here, we used behavioral assays to examine how social isolation at different ages affects emotional discrimination and affiliative helping behavior in mice of both sexes. We found that adult mice raised in social isolation since weaning failed to allogroom distressed conspecifics and were unable to distinguish stressed from unstressed mice in an emotional discrimination assay. Remarkably, 2 weeks of post-weaning social isolation was already sufficient to impair both emotional discrimination and prosocial allogrooming, and these deficits persisted despite 2 months of resocialization. In contrast, 2 weeks of adult social isolation spared emotional discrimination but impaired allogrooming toward stressed conspecifics. These findings reveal a dissociation between emotional discrimination and prosocial helping behavior. Overall, this work identifies adolescence as a sensitive period for the development of emotional state recognition and provides a behavioral framework for investigating how early social experience sculpts neural circuits underlying empathy and stress buffering.

## Significance Statement

Prosocial behaviors are essential for social cohesion and emotional well-being in social animals, but what shapes their development remains poorly understood. We show that social isolation during adolescence impairs the mouse's ability to recognize the emotional state of stressed conspecifics, which cannot be rescued by subsequent resocialization. In contrast, social isolation in adulthood impairs prosocial actions without disrupting emotional discrimination. These findings identify adolescence as a critical period for the development of socio-affective behavior and reveal a dissociation between emotional discrimination and prosocial helping behavior. Our study provides a behavioral framework for investigating how early social experience sculpts neural circuits underlying empathy and stress-buffering and establishes a foundation for mechanistic studies of adolescent vulnerability to social and affective disorders.

## Introduction

Interactions with conspecifics are an essential part of the life of social animals. Many species, from insects to humans, display a rich repertoire of prosocial behaviors, including helping, sharing, and comforting (Penner et al., 2005; Cronin, 2012; Decety et al., 2016; Wu and Hong, 2022). A common feature of these behaviors is that they aim at benefiting other individuals. For instance, primates provide affiliative contact to victims of aggression (Romero et al., 2010), and elephants console distressed companions with trunk touch (Plotnik and de Waal, 2014). Rodents, too, touch and groom those in distress or pain and exhibit rescue-like behaviors toward unresponsive conspecifics (Burkett et al., 2016; Wu et al., 2021; Zhang et al., 2024; F. Sun et al., 2025; W. Sun et al., 2025).

Allogrooming, the act of one animal grooming another conspecific, is one of the best-characterized prosocial behaviors in rodents. It often increases when the conspecific partner is in pain or distress, which makes it a robust, ethologically relevant readout of socio-affective sensitivity. A classic work on prairie voles shows that they groom recently stressed cagemates significantly more than unstressed cagemates; this “consolation-like” behavior depends on oxytocin and buffers the recipient's anxiety and corticosterone levels (Burkett et al., 2016). Similarly, laboratory mouse and rat display consolation-like touch (allogrooming/allolicking) toward distressed familiars, strengthening the case that prosocial touch is a conserved mammalian strategy for social buffering of stress (Lu et al., 2018; Wu et al., 2021). Consistent with a buffering function, stressed rodents that receive allogrooming show reduced anxiety-like behavior compared with those that do not, which links the behavior to measurable improvements in affect (Wu et al., 2021). The ability to recognize and differentiate others' affective states is a prerequisite for appropriately directing prosocial behavior toward individuals in need. This can be assessed in rodents with an emotional discrimination test (Ferretti et al., 2019).

Adolescence is widely recognized as a crucial period for social development. During adolescence, humans acquire social-cognitive skills such as perspective-taking, emotional regulation, and peer interaction strategies, all of which are essential for a successful adult social life (Andrews et al., 2021). Many psychiatric disorders that disrupt social interaction, including depression and schizophrenia, also emerge during adolescence, underscoring its importance in socio-emotional development (Kessler et al., 2007; Paus et al., 2008; Andrews et al., 2021). Insufficient social interaction during adolescence has been linked to hormonal imbalance, depressive symptoms, reduced cognitive capacity, and increased mortality risk (Cacioppo et al., 2015; Holt-Lunstad et al., 2015; Elovainio et al., 2017). The COVID-19 pandemic further highlighted such vulnerability, as prolonged social distancing disproportionately affected children and adolescents, resulting in heightened anxiety and depressive symptoms (Racine et al., 2021; Wang et al., 2022; Gohari et al., 2024). Together, these findings emphasize that adolescent social interaction is essential for healthy socio-emotional development.

In rodents, the post-weaning period, which begins approximately postnatal day (P) 21, corresponds to adolescence and represents a sensitive window for the maturation of social behaviors (Nardou et al., 2019). Social isolation during this period has been widely used as an experimental model to study the consequences of early social deprivation. Previous research has shown that social isolation during adolescence leads to persistent alterations in social behaviors such as aggression and social recognition in adulthood (Kercmar et al., 2011; Li et al., 2021; Takahashi, 2025), but its impact on emotional discrimination and prosocial behavior remains less understood.

In this study, we investigated how social isolation in adolescent mice affects two complementary aspects of social behavior in adulthood: (1) prosocial allogrooming toward stressed conspecifics and (2) emotional discrimination between stressed and unstressed individuals. We found that social isolation from weaning to adulthood impaired both behaviors. Moreover, a brief (2 weeks) social isolation imposed immediately post-weaning sufficed to impair emotional discrimination; the detrimental effects persisted despite prolonged subsequent resocialization. In contrast, 2-week social isolation in adulthood had no detectable effect on emotional discrimination but affected prosocial allogrooming. These results suggest that emotional discrimination and prosocial helping behavior are dissociable and that adolescent social experience is critical for the maturation of socio-emotional functions.

## Materials and Methods

### Experimental animals

C57BL/6J mice (https://www.jax.org/strain/000664) were obtained from The Jackson Laboratory. Both male and female mice were used in this study. Unless otherwise noted, mice were group housed with littermates (2–5 per cage) under standard laboratory conditions [21°C, 50% humidity, 12 h light/dark cycle, zeitgeber time (ZT) 0 = 6:00 A.M.] with *ad libitum* access to food and water. Mice were randomly assigned to experimental groups. All animal procedures were conducted in accordance with the regulations of University of California Santa Cruz Institutional Animal Care and Use Committee (IACUC).

### Social isolation and resocialization

Mice were randomly assigned to social isolation (SI, individual housing in standard cages) or group housing. In adolescent social isolation, mice were isolated at weaning (P21) and remained singly housed for 2 weeks (SI^P21–35^) or until adulthood (∼3 months old, SI^P21-adult^). In adult social isolation, mice were isolated starting on P90 and remained singly housed for 2 weeks (SI^P90–104^).

For resocialization, SI^P21–35^ mice were transferred to a cage containing at least two age- and sex-matched mice that had been group housed together. Most previously isolated mice began sleeping alongside their new cagemates within 1–2 d after resocialization began.

### Prosocial interaction assay

**Figure 1.**
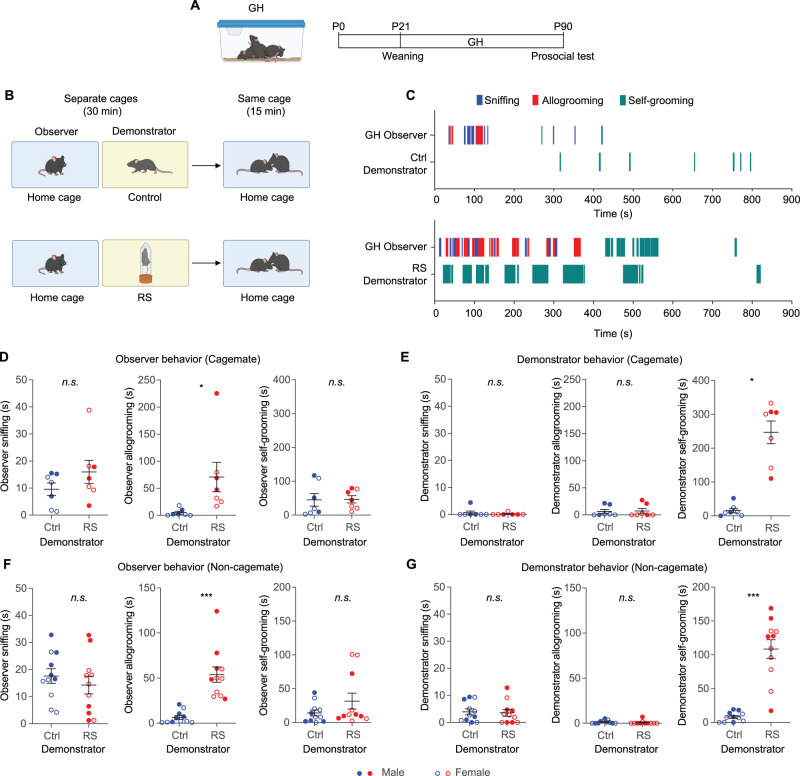
GH adult mice exhibit increased allogrooming toward recently stressed demonstrators. ***A***, Schematic of group housing and timeline of the prosocial test. ***B***, Experimental design for the prosocial test. In the control group, the observer and the demonstrator mouse are separated for 30 min, followed by a 15 min social interaction. In the stress group, the demonstrator is subjected to restraint stress (RS) during the 30 min separation period. ***C***, Representative raster plots illustrating sniffing, self-grooming, and allogrooming of a GH observer and a control (Ctrl) demonstrator (top) or a RS demonstrator (bottom) during social interaction. ***D***, The total time spent on different behaviors by GH observers during interaction with Ctrl versus RS cagemate demonstrators. Sniffing *W* = 12, *p* = 0.3750; allogrooming *W* = 28, *p* = 0.0156; self-grooming *W* = 4, *p* = 0.8125. ***E***, Total time spent on different behaviors by Ctrl versus RS demonstrators during interaction with GH cagemate observers. Sniffing *W* = −1, *p* > 0.9999; allogrooming *W* = 4, *p* = 0.6250; self-grooming *W* = 28, *p* = 0.0156. ***F***, Total time spent on different behaviors by GH observers during interaction with Ctrl versus RS non-cagemate demonstrators. Sniffing *W* = −24, *p* = 0.3203; allogrooming *W* = 66, *p* = 0.0010; self-grooming *W* = 24, *p* = 0.3203. ***G***, Total time spent on different behaviors by Ctrl versus RS demonstrators during interaction with GH non-cagemate observers. Sniffing *W* = −7, *p* = 0.7344; allogrooming *W* = −14, *p* = 0.2969; self-grooming *W* = 66, *p* = 0.0010. Wilcoxon matched-pairs signed rank test for all comparisons. Hereinafter data are presented as mean ± SEM; each data point represents one mouse, with males and females represented by distinct symbols. n.s., not significant; *p < 0.05; **p < 0.01; ***p < 0.001; ****p < 0.0001. Extended Data Figures 1-1 and 1-2 are associated with Figure 1.

The observer and the demonstrator mouse were weight- and sex-matched cagemates (i.e., housed in the same cage since birth) or non-cagemates. Except for experiments in Figure 1*D* and *E*, novel conspecifics were used as demonstrators. All tests were conducted in the observer's home cage. Both observer and demonstrator were habituated to the experimenter's handling for at least 3 d before testing. The observer was also habituated to the test environment by placing its home cage (with the observer only) in the behavioral room for 30 min per day for 3 consecutive days. During these sessions, the observer's cagemates (for group-housed observers) were temporarily transferred to a clean holding cage.

On the test day, both observer and demonstrator cages were transferred to the behavioral room at least 30 min before testing. The observer's cage was placed in the test arena without food or cage lid for 30 min to allow for acclimation. During this period, the control demonstrator was singly housed in a clean cage containing a small amount of bedding from its home cage to minimize novelty-induced stress, while the stressed demonstrator underwent 30 min of restraint stress in a perforated 50 ml conical tube. The observer and the demonstrator were kept in separate rooms during this 30 min separation. The demonstrator was then introduced into the observer's home cage, and social interactions were recorded for 15 min using a CMOS camera (acA1300-60gm, Basler; 1,280 × 960 pixels, 30 Hz frame rate). The same observer always encountered the control demonstrator first and then the stressed demonstrator on the next day. Key behavioral events including sniffing, allogrooming, and self-grooming were manually annotated using BORIS v7.10.2 (Friard and Gamba, 2016). Experiments on cagemates and non-cagemates were conducted using different cohorts of animals.

### Emotion discrimination test

Sex- and age-matched non-cagemate mice served as demonstrators for each observer. For all experiments, novel conspecifics were used as demonstrators. The observer and the demonstrators were habituated to the experimenter's handling prior to testing. The observer was further habituated to the test chamber for 30 min per day for 3 consecutive days before the test. On the test day, both observer and demonstrator cages were transferred to the behavioral room at least 30 min before testing. The emotional discrimination test was conducted in an opaque plexiglass chamber (38 × 28 × 23 cm). Two cylindrical wire cups (height, 10 cm; bottom diameter, 10 cm) were placed in opposite corners of the chamber. The test consisted of three sequential sessions—habituation, baseline, and discrimination—separated by a 30 min stress manipulation between the baseline and the discrimination phase.

In the habituation session, the observer was placed in the chamber containing two empty wire cups for 20 min for acclimation to the test environment. During the baseline session, two demonstrators were placed in the wire cups, and the observer was introduced into the chamber and allowed to interact freely for 5 min.

Following the baseline session, one randomly selected demonstrator was subjected to 30 min of restraint stress, while the other demonstrator remained in a clean cage containing a small amount of bedding from its home cage. During this stress period, the observer was returned to its home cage in a separate room to prevent exposure to auditory, visual, or olfactory cues from demonstrators. In the discrimination session, both demonstrators were returned to their previous wire cups, and the observer was reintroduced into the chamber for 5 min. The chamber and the wire cups were cleaned with 70% ethanol and air-dried between each session. Behaviors were recorded using a CMOS camera (acA1300-60gm, Basler; 1,280 × 960 pixels, 30 Hz frame rate) positioned above the chamber, and the observer's interactions with the wire cups were manually annotated using BORIS v7.10.2. We quantified the interaction number and time with each demonstrator and the discrimination index (DI), defined as $\mathrm{DI}=(T_{\mathrm{animal}\;1}-T_{\mathrm{animal}\;2})/(T_{\mathrm{animal}\;1}+T_{\mathrm{animal}\;2})$, where T denotes the time spent interacting with each demonstrator. We also analyzed the total freezing time of the observer during both baseline and discrimination phases. The freezing behavior refers to the transient immobility of the observer in front of either wired cup containing a demonstrator and was quantified as the total amount of time freezing in front of both cups (control and stressed demonstrators) across both baseline and discrimination phases. This behavior was only observed in SI mice.

### Open field test

The open field test arena was a 44 cm × 44 cm × 44 cm opaque Plexiglas cage. During the test, the subject mouse was placed at the center of the arena and allowed to roam freely and uninterrupted for 10 min. Behavior was recorded using a CMOS camera (acA1300-60gm, Basler; 1,280 × 960 pixels, 30 Hz frame rate) and analyzed with MouseActivity, an open-source MATLAB program (Zhang et al., 2020). Distance traveled and time spent in the arena center versus periphery were measured following the protocol described previously (Zhang et al., 2020).

### Statistics

All behavioral data were analyzed with the analyst blinded to experimental conditions. All statistical analyses were performed with GraphPad Prism 10 (GraphPad Software). We used Mann–Whitney test to compare two independent samples, Wilcoxon matched-pairs signed rank test to compare two paired samples, and two-sided one-sample *t* test to compare the mean of a sample to a specified value. To compare more than two independent samples, we used one-way ANOVA followed by Tukey's multiple-comparisons test if the samples passed normality test; otherwise, we used Kruskal–Wallis test followed by Dunn's multiple-comparisons test. To determine the effect of two independent factors (e.g., demonstrator status and test phase) with repeated measures on the same subject, we used two-way repeated-measures ANOVA followed by uncorrected Fisher's LSD post hoc test. The sample size *n* refers to the number of mice. We indicated the sex of individual animals by distinct symbols for males and females in figures wherever appropriate. We report the statistical tests used and the *p* values in the figure legends. Statistical significance is defined as *p* < 0.05.

## Results

### Mice perform prosocial allogrooming toward stressed conspecifics, independent of sex or familiarity

Allogrooming, the act of one animal grooming another, is a key component of social behavior, serving both affiliative and consoling functions (Wu et al., 2021). We asked whether mice would preferentially direct this behavior toward stressed conspecifics. We started by separating pairs of adult mice (∼3 months old) that had been reared together since birth (group housed or GH). One mouse was designated as the “observer” and the other as the “demonstrator.” While the observer stayed in the home cage, the demonstrator was temporarily removed from the home cage and either placed in another empty cage (control demonstrator) or subjected to 30 min restraint stress (RS demonstrator). The demonstrator was then returned to the home cage, and interactions between the observer and the demonstrator were recorded for 15 min (Fig. 1*A*,*B*).

10.1523/ENEURO.0441-25.2026.f1-1 Figure 1-1 Behavioral analyses of observers and demonstrators during social interaction under different conditions. ***A,*** Observer mice show significantly more sniffing toward cagemate Ctrl demonstrators than vice versa. Wilcoxon matched-pairs signed rank test, *W* = -28, *p* = 0.0156. ***B,*** Observer mice show significantly more sniffing toward cagemate RS demonstrators than vice versa. Wilcoxon matched-pairs signed rank test, *W* = -28, *p* = 0.0156. ***C***, Observer mice exhibit comparable amount of allogrooming toward cagemate vs non-cagemate RS demonstrators. Mann-Whitney test: *U* = 37, *p* = 0.9298. ***D*,** Cagemate RS demonstrators show more self-grooming than non-cagemate RS demonstrators during the prosocial test. Mann-Whitney test, *U* = 8, *p* = 0.0041. ***E,*** Allogrooming of male and female observers toward non-cage demonstrators. Two-way repeated measures ANOVA, main effect of demonstrator status: *F*(1,9) = 35.16, *p* = 0.0002; main effect of sex: *F*(1,9) = 3.411, *p* = 0.0978; interaction between demonstrator status and sex: *F*(1,9) = 1.532, *p* = 0.2471. Uncorrected Fisher’s LSD: male (Ctrl vs RS demonstrators) *p* = 0.0009; female (Ctrl vs RS demonstrator) *p* = 0.0069. ***F,*** Self-grooming of male and female demonstrators in present of non-cage observers. Two-way repeated measures ANOVA, main effect of demonstrator status: *F*(1,9) = 59.99, *p* < 0.0001; main effect of sex: *F*(1,9) = 0.6936, *p* = 0.4265; interaction between demonstrator status and sex: *F*(1,9) = 0.2199, *p* = 0.6555. Uncorrected Fisher’s LSD: male (Ctrl vs RS demonstrators) *p* = 0.0004; female (Ctrl vs RS demonstrators) *p* = 0.0004. Download Figure 1-1, TIF file.

**Figure 2.**
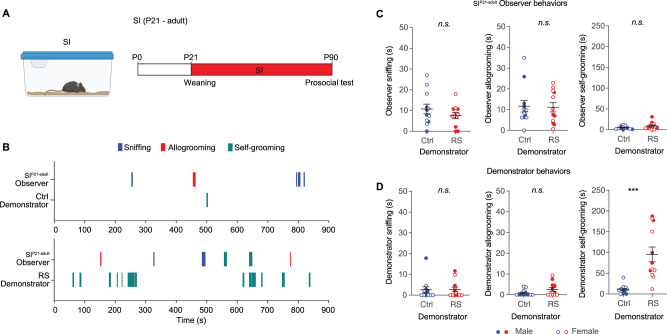
SI^P21-adult^ mice do not exhibit increased allogrooming toward stressed demonstrators. ***A***, A diagram illustrating the housing condition and the experimental timeline. ***B***, Representative raster plots illustrating sniffing, self-grooming, and allogrooming of a SI^P21-adult^ observer and a Ctrl demonstrator (top) or a RS demonstrator (bottom) during social interaction. ***C***, Total time spent on different behaviors by SI^P21-adult^ observers during interaction with Ctrl versus RS demonstrators. Sniffing *W* = −21, *p* = 0.3770; allogrooming *W* = 4, *p* = 0.8945; self-grooming *W* = 33, *p* = 0.2109. ***D***, Total time spent on different behaviors by Ctrl versus RS demonstrators during interaction with SI^P21-adult^ observers. Sniffing *W* = −2, *p* = 0.9453; allogrooming *W* = 23, *p* = 0.2031; self-grooming *W* = 66, *p* = 0.0010. Wilcoxon matched-pairs signed rank test for all comparisons. Extended Data Figures 2-1 and 2-2 are associated with Figure 2.

10.1523/ENEURO.0441-25.2026.f1-2 Figure 1-2 Allogrooming reduces the anxiety of RS demonstrators as assessed by the open field test. ***A*,** Schematic of the open field test. ***B*,** Movement trajectory of a control mouse over 15 min. ***C*,** Movement trajectory of a mouse recently subjected to 30 min RS following by 15 min single-housing recovery. ***D***, Movement trajectory of a mouse recently subjected to 30 min RS followed by 15 min of prosocial interaction. ***E*,** Total distance traveled for all the experimental groups. One-way ANOVA, *F*(2, 18) = 0.2904, *p* = 0.7514. ***F,*** Fraction of time spent in the peripheral region of the open field for all experimental groups. One-way ANOVA, *F*(2, 18) = 5.360, *p* = 0.0149; Tukey’s multiple comparisons test: Ctrl vs RS *p* = 0.0387; Ctrl vs RS + prosocial *p* = 0.9554; RS vs RS + prosocial *p* = 0.0214. *n* = 7 female mice. Download Figure 1-2, TIF file.

We quantified three types of behaviors during observer–demonstrator interactions: anogenital sniffing, self-grooming, and allogrooming (Movies 1–3). We found that observers spent similar amount of time sniffing or self-grooming regardless of demonstrator status (Fig. 1*D*). In contrast, they spent significantly more time allogrooming RS demonstrators than control demonstrators (Fig. 1*C*,*D*). On the other hand, both types of demonstrators showed little allogrooming (Fig. 1*E*) and sniffed observers less frequently than observers sniffed them (Extended Data Fig. 1-1*A,B*). Notably, RS demonstrators self-groomed markedly more than controls (Fig. 1*C*,*E*), consistent with stress-induced anxiety (Fernandez-Teruel and Estanislau, 2016).

**Movie 1.** An example of sniffing behavior. [View online]

**Movie 2.** An example of self-grooming. [View online]

**Movie 3.** An example of allogrooming. [View online]

To determine whether the increase in allogrooming toward stressed conspecifics depends on social familiarity, we repeated the same assay with non-cagemate demonstrators. Observers displayed minimal allogrooming toward control non-cagemate demonstrators but significantly more toward RS non-cagemates (Fig. 1*F*). The amount of observer's allogrooming toward RS non-cagemates was comparable with that toward RS cagemates (Extended Data Fig. 1-1*C*). However, cagemate RS demonstrators self-groomed significantly more than non-cagemate RS demonstrators did (Extended Data Fig. 1-1*D*). Both observer's allogrooming and demonstrator's self-grooming were comparable between male and female pairs (Extended Data Fig. 1-1*E,F*). Thus, the increase in allogrooming is contingent upon the demonstrator's stress state, but independent of the observer's sex or prior social familiarity with the demonstrator.

Next, we investigated whether the demonstrator's self-grooming served as a cue for observer allogrooming by examining their temporal relationship. We found that demonstrator self-grooming was not a prerequisite for the initiation of observer allogrooming. Specifically, 14 of 18 observers initiated at least one bout of allogrooming before the demonstrator exhibited any self-grooming (80 of 246 total allogrooming bouts). This occurred in 4 of 7 observers with cagemate demonstrators (11 of 86 bouts) and 10 of 11 observers with non-cagemate demonstrators (69 of 160 bouts). Furthermore, only a small fraction of observer allogrooming bouts were initiated during demonstrator self-grooming: 30 of 246 episodes total (12.2%), with similar rates across cagemate (11 of 86 bouts, 12.8%) and non-cagemate (19 of 160 bouts, 11.9%) demonstrator groups. We also quantified how often observer allogrooming occurred shortly after demonstrator self-grooming onset. Only 28 of 246 observer allogrooming bouts (11.4%) occurred within 5 s, and 47 of 246 bouts (19.1%) within 10 s, of demonstrator self-grooming onset. Moreover, the average latency between the onset of observer allogrooming and the onset of the most recent demonstrator self-grooming bout exceeded 1 min (64.9 s for cagemate demonstrators and 62.6 s for non-cagemate demonstrators). Collectively, these data suggest that demonstrator self-grooming is neither a necessary nor a primary cue driving observer allogrooming or emotional discrimination.

Finally, we examined whether allogrooming would alleviate stress-induced anxiety in demonstrators. Adult mice were subjected to 30 min RS and then either singly housed for 15 min or paired with another mouse for 15 min to allow allogrooming. Anxiety-like behavior was subsequently assessed using the open field test. Compared with unstressed controls, RS mice traversed similar distances but spent significantly more time in the periphery of the arena. However, RS demonstrators that received allogrooming spent similar amount of time in the center of the arena as unstressed controls (Extended Data Fig. 1-2).

Together, these results indicate that mice of both sexes exhibit prosocial allogrooming toward stressed conspecifics, independent of prior familiarity, which alleviates stress-induced anxiety of the recipient.

### Social isolation since weaning impairs prosocial allogrooming

Next, we asked how early social experience shapes adult prosocial behavior. We reared mice either in social isolation (SI) from weaning (P21) or with standard GH (Fig. 2*A*) and examined their prosocial behavior in adulthood (P90). We found that SI^P21-adult^ observers behaved similarly as GH observers when interacting with control demonstrators (sniffing, allogrooming, and self-grooming; Extended Data Fig. 2-1*A–C*). However, in the presence of RS demonstrators, SI^P21-adult^ observers showed normal sniffing (Fig. 2*C*, Extended Data Fig. 2-1*D*) but did not exhibit increased allogrooming as GH observers (Fig. 2*C*, Extended Data Fig. 2-1*E*). As expected, RS demonstrators self-groomed more than controls, regardless of whether their partner was a GH or SI^P21-adult^ observer (compare Figs. 2*D*, 1*G*). Furthermore, both observer's allogrooming and demonstrator's self-grooming were comparable between male and female pairs (Extended Data Fig. 2-2). Taken together, these findings indicate that early social experience is critical for the development of prosocial grooming.

10.1523/ENEURO.0441-25.2026.f2-1 Figure 2-1 Behavioral comparison between adult GH and SI^P21-adult^ observers toward Ctrl vs RS demonstrators. ***A***, Adult GH and SI^P21-adult^ mice show comparable sniffing toward Ctrl demonstrators. *U* = 40, *p* = 0.1179. ***B***, Adult GH and SI^P21-adult^ mice show comparable allogrooming toward Ctrl demonstrators. *U* = 39, *p* = 0.1002. ***C***, Adult GH and SI^P21-adult^ mice show comparable self-grooming during interaction with Ctrl demonstrators. *U* = 44, *p* = 0.1839. ***D***, Adult GH and SI^P21-adult^ mice show comparable sniffing toward RS demonstrators. *U* = 44, *p* = 0.1841. ***E***, Adult GH mice exhibit significantly more allogrooming than SI^P21-adult^ mice toward RS demonstrators. *U* = 0, *p* < 0.0001. ***F***, Adult GH and SI^P21-adult^ mice show comparable self-grooming during interaction with RS demonstrators. *U* = 41, *p* = 0.1301. Mann-Whitney test for all comparisons. Download Figure 2-1, TIF file.

10.1523/ENEURO.0441-25.2026.f2-2 Figure 2-2 Both male and female SI^P21-adult^ observers show impaired allogrooming despite enhanced self-grooming of RS demonstrators. ***A,*** Allogrooming of male and female SI^P21-adult^ observers toward Ctrl and RS demonstrators. Two-way repeated measures ANOVA, main effect of demonstrator status: *F*(1,10) = 0.07436, *p* = 0.7906; main effect of sex: *F*(1,10) = 0.3592, *p* = 0.5623; interaction between demonstrator status and sex: *F*(1,10) = 0.8038, *p* = 0.3910. ***B,*** Self-grooming of male and female demonstrators in the presence of SI^P21-adult^ observers. Two-way repeated measures ANOVA, main effect of demonstrator status: *F*(1,10) = 22.18, *p* = 0.0008; main effect of sex: *F*(1,10) = 0.7383, *p* = 0.4103; interaction between demonstrator status and sex: *F*(1,10) = 1.850, *p* = 0.2037. Uncorrected Fisher’s LSD: male (Ctrl vs RS demonstrators) *p* = 0.0026; female (Ctrl vs RS demonstrators) *p* = 0.0267. Download Figure 2-2, TIF file.

### Social isolation since weaning impairs emotional discrimination

**Figure 3.**
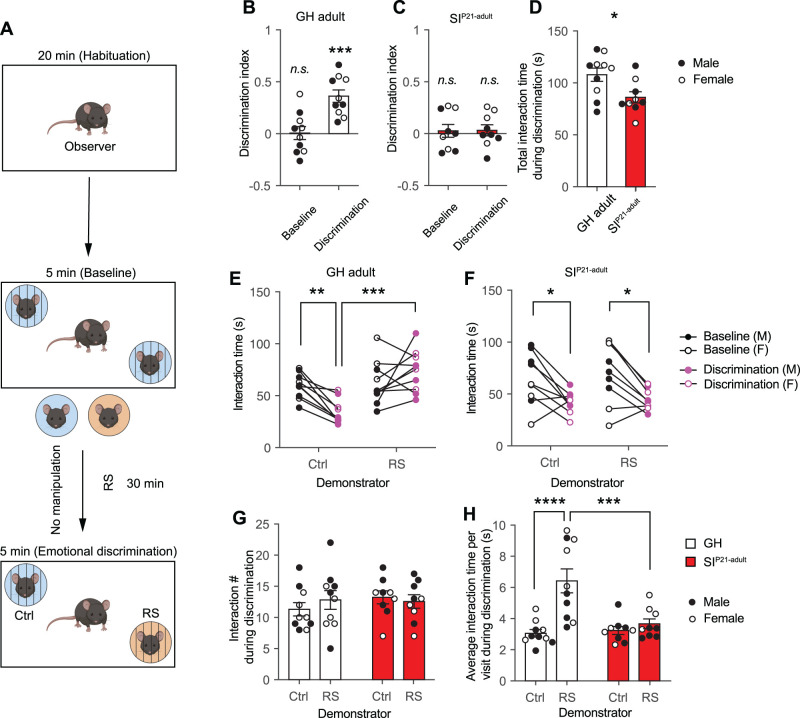
Adult GH mice preferentially interact with recently stressed demonstrators in an emotional discrimination test, while SI^P21-adult^ mice fail to do so. ***A***, Experimental setup of the emotional discrimination test. ***B***, Adult GH mice preferentially interact with RS mice during discrimination. The discrimination index $\mathrm{DI}=(T_{\mathrm{RS}}-T_{\mathrm{Ctrl}})/(T_{\mathrm{RS}}+T_{\mathrm{Ctrl}})$, T denotes the time spent interacting with the demonstrator. The demonstrator is designated Ctrl or RS based on its status during discrimination. One-sample *t* test compared with 0: baseline *t*_(9)_ = 0.0913, *p* = 0.9292; discrimination *t*_(9)_ = 6.063, *p* = 0.0002. ***C***, Adult SI^P21-adult^ mice do not show interaction preference during discrimination. One-sample *t* test compared with 0: baseline *t*_(8)_ = 0.4339, *p* = 0.6759; discrimination *t*_(8)_ = 0.5988, *p* = 0.5659. ***D***, Total interaction time during discrimination in GH versus SI^P21-adult^ mice. Mann–Whitney test, *U* = 17, *p* = 0.0220. ***E***, Interaction time of GH adult with Ctrl versus RS demonstrators during baseline and discrimination. Two-way repeated-measures ANOVA, main effect of demonstrator status: *F*_(1,18)_ = 13.69, *p* = 0.0016; main effect of experiment phase: *F*_(1,18)_ = 2.136, *p* = 0.1611; interaction between demonstrator status and experiment phase: *F*_(1,18)_ = 10.58, *p* = 0.0044. Uncorrected Fisher's LSD: interaction with Ctrl demonstrator (baseline vs discrimination) *p* = 0.0015; interaction with RS demonstrator (baseline vs discrimination) *p* = 0.1155; baseline interaction (Ctrl vs RS demonstrators) *p* = 0.7554; discrimination phase interaction (Ctrl vs RS demonstrators) *p* = 0.0001. ***F***, Interaction time of SI^P21-adult^ mice with Ctrl and RS demonstrators during baseline and discrimination. Two-way repeated-measures ANOVA, main effect of demonstrator status: *F*_(1,16)_ = 0.6393, *p* = 0.4357; main effect of experiment phase: *F*_(1,16)_ = 8.166, *p* = 0.0114; interaction between demonstrator status and experiment phase: *F*_(1,16)_ = 0.0898, *p* = 0.7684. Uncorrected Fisher's LSD: interaction with Ctrl demonstrator (baseline vs discrimination) *p* = 0.0262; interaction with RS demonstrator (baseline vs discrimination) *p* = 0.0130. ***G***, Total interaction number of GH and SI^P21-adult^ mice toward Ctrl versus RS demonstrators during discrimination. Two-way repeated-measures ANOVA, main effect of demonstrator status: *F*_(1,17)_ = 0.1586, *p* = 0.6954; main effect of observer housing condition: *F*_(1,17)_ = 0.3892, *p* = 0.5410; interaction between demonstrator status and observer housing condition: *F*_(1,17)_ = 1.072, *p* = 0.3149. ***H***, Average interaction time per visit of GH and SI^P21-adult^ mice toward Ctrl versus RS demonstrators during discrimination. Two-way repeated-measures ANOVA, main effect of demonstrator status: *F*_(1,17)_ = 21.58, *p* = 0.0002; main effect of observer housing condition: *F*_(1,17)_ = 6.089, *p* = 0.0245; interaction between demonstrator status and observer housing condition: *F*_(1,17)_ = 12.93, *p* = 0.0022. Uncorrected Fisher's LSD: interaction with Ctrl demonstrator (GH vs SI^P21-adult^) *p* = 0.7858; interaction with RS demonstrator (GH vs SI^P21-adult^) *p* = 0.0002; GH observer interaction (Ctrl vs RS demonstrators) *p* < 0.0001; SI^P21-adult^ observer interaction (Ctrl vs RS demonstrators) *p* = 0.4797. Extended Data Figure 3-1 is associated with Figure 3.

To determine whether impaired allogrooming in SI^P21-adult^ mice reflects deficits in recognizing conspecific stress, we performed an emotional discrimination test (Ferretti et al., 2019; Fig. 3*A*). In this test, an observer mouse was presented with two age- and sex-matched non-cagemate demonstrator mice confined in wired cups. After a 5 min baseline phase, one demonstrator was temporarily moved to a new empty cage (control demonstrator) and the other underwent 30 min restraint stress (RS demonstrator). In the discrimination phase, both demonstrators were returned to the wired cups and the observer freely interacted with them for another 5 min. We measured the total interaction time with each demonstrator and calculated the discrimination index (DI), defined as $\mathrm{DI}=(T_{\mathrm{RS}}-T_{\mathrm{Ctrl}})/(T_{\mathrm{RS}}+T_{\mathrm{Ctrl}})$, where T denotes the time spent interacting with each demonstrator.

10.1523/ENEURO.0441-25.2026.f3-1 Figure 3-1 Behavior of GH adults and SI^P21-adult^ observers during the emotional discrimination test. ***A***, Discrimination index of male and female adult GH observers. Two-way repeated measures ANOVA, main effect of sex: *F*(1,8) = 0.3170, *p* = 0.5889; main effect of experiment phase: *F*(1,8) = 27.58, *p* = 0.0008; interaction between sex and experiment phase: *F*(1,8) = 1.121, *p* = 0.3207. Uncorrected Fisher’s LSD: male (baseline vs discrimination) *p* = 0.0021; female (baseline vs discrimination) *p* = 0.0180. ***B***, Interaction time of male and female adult GH observers. Ctrl demonstrator: Two-way repeated measures ANOVA, main effect of sex: *F*(1,8) = 0.4931, *p* = 0.5025; main effect of experiment phase: *F*(1,8) = 33.06, *p* = 0.0004; interaction between sex and experiment phase: *F*(1,8) = 0.1101, *p* = 0.7485. Uncorrected Fisher’s LSD: male (baseline vs discrimination) *p* = 0.0026; female (baseline vs discrimination) *p* = 0.0050. RS demonstrator: Two-way repeated measures ANOVA, main effect of sex: *F*(1,8) = 2.351, *p* = 0.1637; main effect of experiment phase: *F*(1,8) = 2.030, *p* = 0.1920; interaction between sex and experiment phase: *F*(1,8) = 0.6178, *p* = 0.4545. ***C***, Discrimination index of male and female SI^P21-adult^ observers. Two-way repeated measures ANOVA, main effect of sex: *F*(1,7) = 2.829, *p* = 0.1365; main effect of experiment phase: *F*(1,7) = 0.02022, *p* = 0.8909; interaction between sex and experiment phase: *F*(1,7) = 0.3944, *p* = 0.5499. ***D***, Interaction time of male and female SI^P21-adult^ observers. Ctrl demonstrator: Two-way repeated measures ANOVA, main effect of sex: *F*(1,7) = 9.211, *p* = 0.0190; main effect of experiment phase: *F*(1,7) = 7.942, *p* = 0.0258; interaction between sex and experiment phase: *F*(1,7) = 1.943, *p* = 0.2060. Uncorrected Fisher’s LSD: male (baseline vs discrimination) *p* = 0.0160; female (baseline vs discrimination) *p* = 0.3711; baseline (male vs female) *p* = 0.0080; discrimination (male vs female) *p* = 0.3127. RS demonstrator: Two-way repeated measures ANOVA, main effect of sex: *F*(1,7) = 0.06564, *p* = 0.8052; main effect of experiment phase: *F*(1,7) = 11.31, *p* = 0.0120; interaction between sex and experiment phase: *F*(1,7) = 1.064, *p* = 0.3367. Uncorrected Fisher’s LSD: male (baseline vs discrimination) *p* = 0.0132; female (baseline vs discrimination) *p* = 0.1618. Download Figure 3-1, TIF file.

During the baseline phase, both adult GH and SI^P21-adult^ observers spent comparable time with the two demonstrators, yielding DI values near zero (Fig. 3*B*,*C*). In the discrimination phase, GH observers preferentially interacted with the RS demonstrator (DI significantly greater than zero; Fig. 3*B*), whereas SI^P21-adult^ observers showed no preference (DI ≈ 0; Fig. 3*C*). Total interaction time during discrimination was lower in SI^P21-adult^ than GH observers (Fig. 3*D*). Moreover, we found that GH observers spent significantly less time interacting with control demonstrators during discrimination than baseline but comparable time with RS demonstrators during the two phases (Fig. 3*E*; note that we labeled a demonstrator as “RS” even during the baseline, if it underwent RS prior to the discrimination phase). In contrast, SI^P21-adult^ mice spent less time interacting with demonstrators during discrimination than at the baseline, irrespective of whether the demonstrator was stressed or not (Fig. 3*F*). The number of interaction bouts was similar across groups and demonstrator status (Fig. 3*G*). The preference of GH observers to interact with RS demonstrators during discrimination arose from longer bouts on average rather than more frequent approaches (Fig. 3*H*). While GH mice showed no sex difference in either the discrimination index or interaction time (Extended Data Fig. 3-1*A,B*), SI^P21-adult^ mice exhibited a weak sex difference in interaction time, but not in the discrimination index (Extended Data Fig. 3-1*C,D*). These results show that SI^P21-adult^ mice fail to discriminate between stressed and control conspecifics.

### Brief social isolation during adolescence suffices to impair emotional discrimination and allogrooming

**Figure 4.**
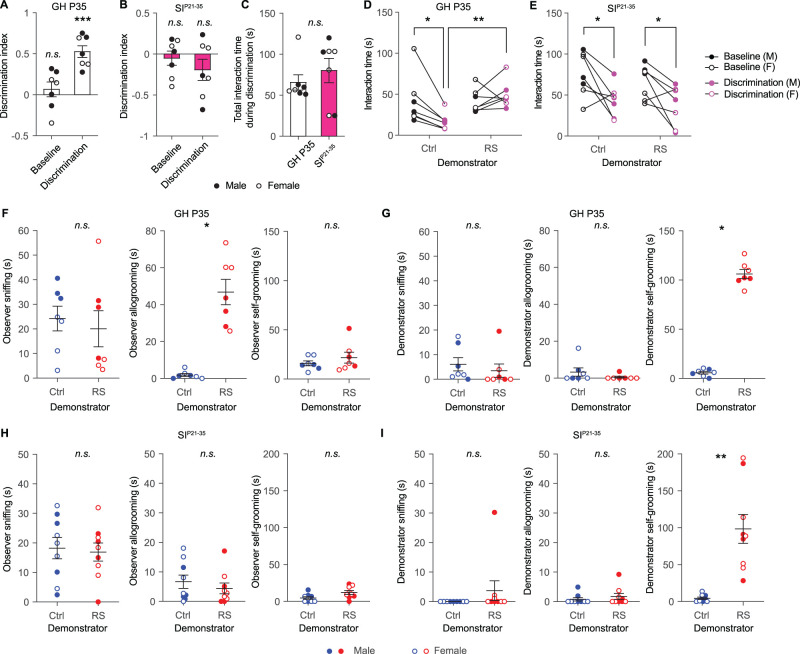
Two-week social isolation starting from weaning is sufficient to impair emotional discrimination and prosocial allogrooming. ***A***, P35 GH mice preferentially interact with RS demonstrators during the discrimination phase. One-sample *t* test compared with 0: baseline *t*_(6)_ = 0.7171, *p* = 0.5002; discrimination *t*_(6)_ = 7.302, *p* = 0.0003. ***B***, SI^P21–35^ mice do not show interaction preference during discrimination. One-sample *t* test compared with 0: baseline *t*_(6)_ = 0.5858, *p* = 0.5794; discrimination *t*_(6)_ = 1.497, *p* = 0.1851. ***C***, Total interaction time during discrimination is comparable between GH and SI^P21–35^ mice at P35. Mann–Whitney test, *U* = 19, *p* = 0.5350. ***D***, Interaction time of P35 GH with Ctrl versus RS observers during baseline and discrimination. Two-way repeated-measures ANOVA, main effect of demonstrator status: *F*_(1,12)_ = 9.306, *p* = 0.0101; main effect of experiment phase: *F*_(1,12)_ = 1.031, *p* = 0.3299; interaction between demonstrator status and experiment phase: *F*_(1,12)_ = 9.132, *p* = 0.0106. Uncorrected Fisher's LSD: interaction with Ctrl demonstrator (baseline vs discrimination) *p* = 0.0215; interaction with RS demonstrator (baseline vs discrimination) *p* = 0.4692; baseline interaction (Ctrl vs RS demonstrators) *p* = 0.9842; discrimination phase interaction (Ctrl vs RS demonstrators) *p* = 0.0010. ***E***, Interaction time between SI^P21–35^ mice and demonstrators (both Ctrl and RS) is less during discrimination than during baseline. Two-way repeated-measures ANOVA, main effect of demonstrator status: *F*_(1,12)_ = 1.101, *p* = 0.3148; main effect of experiment phase: *F*_(1,12)_ = 9.524, *p* = 0.0094; interaction between demonstrator status and experiment phase: *F*_(1,12)_ = 0.041, *p* = 0.8429. Uncorrected Fisher's LSD: interaction with Ctrl demonstrator (baseline vs discrimination) *p* = 0.0159; interaction with RS demonstrator (baseline vs discrimination) *p* = 0.0271; baseline interaction (Ctrl vs RS demonstrators) *p* = 0.3935; discrimination phase interaction (Ctrl vs RS demonstrators) *p* = 0.5605. ***F***, Total time spent on different behaviors by P35 GH observers during interaction with Ctrl versus RS demonstrators. Sniffing *W* = −14, *p* = 0.2656; allogrooming *W* = 28, *p* = 0.0156; self-grooming *W* = 6, *p* = 0.6875. ***G***, Total time spent on different behaviors by Ctrl versus RS demonstrators during interaction with P35 GH observers. Sniffing *W* = −9, *p* = 0.5000; allogrooming *W* = −6, *p* = 0.3750; self-grooming *W* = 28, *p* = 0.0156. ***H***, Total time spent on different behaviors by SI^P21–35^ observers during interaction with Ctrl versus RS demonstrators. Sniffing *W* = −15, *p* = 0.4102; allogrooming *W* = −11, *p* = 0.5703; self-grooming *W* = 25, *p* = 0.1641. ***I***, Total time spent on different behaviors by Ctrl versus RS demonstrators during interaction with SI^P21–35^ observers. Sniffing *W* = 6, *p* = 0.2500; allogrooming *W* = 8, *p* = 0.2500; self-grooming *W* = 45, *p* = 0.0039. Wilcoxon matched-pairs signed rank test for all comparisons in ***F–I***. Extended Data Figure 4-1 is associated with Figure 4.

We next investigated how the timing of social isolation affects emotional discrimination. To assess the effect of adolescent isolation, we raised mice either under GH or under SI for 2 weeks starting at weaning (SI^P21–35^) and then tested them at P35 using the emotional discrimination assay. During the baseline phase, both GH and SI^P21–35^ mice showed comparable, approximately zero DI (Fig. 4*A*,*B*). However, during the discrimination phase, GH observers exhibited a significantly positive DI, reflecting a clear preference for interacting with the RS demonstrator (Fig. 4*A*), whereas SI^P21–35^ observers showed no such preference (Fig. 4*B*). Total interaction time during the discrimination phase did not differ between the two observer groups (Fig. 4*C*). Comparing the interaction time with the same mouse during baseline and discrimination, P35 GH mice, like adult GH mice, showed a decrease in interaction with control demonstrators but not RS demonstrators (Fig. 4*D*). However, SI^P21–35^ mice exhibited reduced interaction with both control and RS demonstrators (Fig. 4*E*). P35 GH mice showed an increase in both interaction number and average interaction time toward RS demonstrators during the discrimination phase, both of which were impaired in the SI^P21–35^ mice (Extended Data Fig. 4-1). Furthermore, unlike GH P35 mice, SI^P21–35^ mice failed to increase allogrooming toward RS demonstrators (Fig. 4*F*–*I*). Together, these results suggest that 2-week adolescent social isolation is sufficient to impair the normal development of emotional discrimination and prosocial behavior.

10.1523/ENEURO.0441-25.2026.f4-1 Figure 4-1 Behavior of P35 GH and SI^P21-35^ observers during the discrimination phase of the emotional discrimination test. ***A***, Total interaction number of P35 GH and SI^P21-35^ mice toward Ctrl vs RS demonstrators during discrimination. Two-way repeated measures ANOVA, main effect of demonstrator status: *F*(1,12) = 7.763, *p* = 0.0165; main effect of observer housing condition: *F*(1,12) = 0.2656, *p* = 0.6157; interaction between demonstrator status and observer housing condition: *F*(1,12) = 2.077, *p* = 0.1751. Uncorrected Fisher’s LSD: GH observer interaction (Ctrl vs RS demonstrators) *p* = 0.0113; SI^P21-35^ observer interaction (Ctrl vs RS demonstrators) *p* = 0.3603. ***B***, Average interaction time per visit of P35 GH and SI^P21-35^ mice toward Ctrl vs RS demonstrators during discrimination. Two-way repeated measures ANOVA, main effect of demonstrator status: *F*(1,12) = 2.951, *p* = 0.1115; main effect of observer housing condition: *F*(1,12) = 4.234, *p* = 0.0620; interaction between demonstrator status and observer housing condition: *F*(1,12) = 32.82, *p* < 0.0001. Uncorrected Fisher’s LSD: interaction with Ctrl demonstrator (GH vs SI^P21-35^ observers) *p* = 0.0002; interaction with RS demonstrator (GH vs SI^P21-35^ observers) *p* = 0.5231; GH observer interaction (Ctrl vs RS demonstrators) *p* = 0.0002; SI^P21-35^ observer interaction (Ctrl vs RS demonstrators) *p* = 0.015. Download Figure 4-1, TIF file.

### Brief social isolation during adulthood spares emotional discrimination but impairs prosocial allogrooming

**Figure 5.**
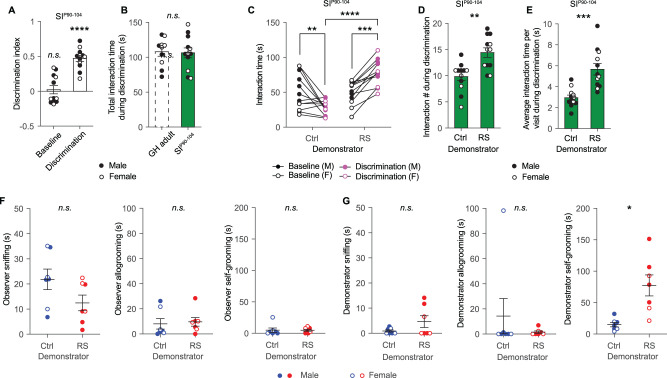
Two-week SI in adulthood does not affect emotional discrimination but impairs prosocial allogrooming. ***A***, SI^P90–104^ mice preferentially interact with RS mice during discrimination. One-sample *t* test compared with 0: baseline *t*_(11)_ = 0.3506, *p* = 0.7325; discrimination *t*_(11)_ = 10.97, *p* < 0.0001. ***B***, Total interaction time during discrimination is comparable between adult GH and SI^P90–104^ mice. Mann–Whitney test, *U* = 53, *p* = 0.6744. GH data are the same as in Figure 3*D*. ***C***, Interaction time of SI^P90–104^ mice with Ctrl versus RS demonstrators during baseline and discrimination. Two-way repeated-measures ANOVA, main effect of demonstrator status: *F*_(1,22)_ = 40.03, *p* < 0.0001; main effect of experiment phase: *F*_(1,22)_ = 0.2883, *p* = 0.5967; interaction between demonstrator status and experiment phase: *F*_(1,22)_ = 42.62, *p* < 0.0001. Uncorrected Fisher's LSD: interaction with Ctrl demonstrator (baseline vs discrimination) *p* = 0.0066; interaction with RS demonstrator (baseline vs discrimination) *p* = 0.0005; baseline interaction (Ctrl vs RS demonstrator) *p* = 0.8879; discrimination phase interaction (Ctrl vs RS demonstrator) *p* < 0.0001. ***D***, SI^P90–104^ mice interact more frequently with RS demonstrators than Ctrl demonstrators during discrimination. *W* = 73, *p* = 0.0020. ***E***, SI^P90–104^ mice show significantly longer average interaction time per visit toward RS demonstrators than Ctrl demonstrators during discrimination. *W* = 78, *p* = 0.0005. ***F***, Total time spent on different behaviors by SI^P90–104^ observers during interaction with Ctrl versus RS demonstrators. Sniffing *W* = −22, *p* = 0.0781; allogrooming *W* = 6, *p* = 0.6875; self-grooming *W* = −1, *p* > 0.9999. ***G***, Total time spent on different behaviors by Ctrl versus RS demonstrators during interaction with SI^P90–104^ observers. Sniffing *W* = 9, *p* = 0.3125; allogrooming *W* = 2, *p* = 0.8750; self-grooming *W* = 28, *p* = 0.0156. Wilcoxon matched-pairs signed rank test for ***D–G***. Extended Data Figure 5-1 is associated with Figure 5.

To determine whether social isolation has similar effects in adulthood, we socially isolated mice for 2 weeks beginning at P90 (SI^P90–104^) and then tested them using the same assay. SI^P90–104^ observers preferentially interacted with RS demonstrators during discrimination (DI significantly greater than zero; Fig. 5*A*), with a total interaction time comparable with that of GH adults (Fig. 5*B*). Just as GH adults, SI^P90–104^ observers interacted with control demonstrators less during discrimination than baseline (Fig. 5*C*). However, unlike SI^P21–35^ and SI^P21-adult^ mice, which show decreased interaction with RS demonstrators during discrimination (Figs. 4*E*, 3*F*), SI^P90–104^ mice show increased interaction with RS demonstrators during discrimination (Fig. 5*C*). Although this pattern might suggest that SI^P90–104^ mice have stronger differential responses to stressed versus non-stressed conspecifics, direct comparison between adult GH and SI^P90–104^ mice revealed no significant difference in either interaction time or DI (Extended Data Fig. 5-1). Unlike in GH adults, the increase in DI during the discrimination phase exhibited by SI^P90–104^ mice was driven by both longer interaction time per visit and more frequent interactions (Fig. 5*D*,*E*). Surprisingly, SI^P90–104^ mice failed to increase allogrooming toward RS demonstrators (Fig. 5*F*), despite elevated self-grooming of RS demonstrators (Fig. 5*G*). Thus, adolescent social isolation disrupts both emotional discrimination and prosocial allogrooming, whereas adult isolation selectively impairs prosocial allogrooming without abolishing emotional discrimination.

10.1523/ENEURO.0441-25.2026.f5-1 Figure 5-1 Behavior of adult GH and SI^P90-104^ observers during the emotional discrimination test. ***A***, Interaction time during baseline. Two-way repeated measures ANOVA, main effect of demonstrator status: *F*(1,20) = 0.03242, *p* = 0.8589; main effect of observer housing condition: *F*(1,20) = 2.586, *p* = 0.1235; interaction between demonstrator status and observer housing condition: *F*(1,20) = 0.1132, *p* = 0.7400. ***B***, Interaction time during discrimination. Two-way repeated measures ANOVA, main effect of demonstrator status: *F*(1,20) = 97.77, *p* < 0.0001; main effect of observer housing condition: *F*(1,20) = 0.01804, *p* = 0.8945; interaction between demonstrator status and observer housing condition: *F*(1,20) = 1.139, *p* = 0.2985. Uncorrected Fisher’s LSD: GH observer (Ctrl vs RS demonstrators) *p* < 0.0001; SI^P90-104^ observer (Ctrl vs RS demonstrators) *p* < 0.0001. ***C***, Discrimination index. Two-way repeated measures ANOVA, main effect of experiment phase: *F*(1,20) = 74.09, *p* < 0.0001; main effect of observer housing condition: *F*(1,20) = 0.9367, *p* = 0.3447; interaction between experiment phase and observer housing condition: *F*(1,20) = 1.005, *p* = 0.3280. Uncorrected Fisher’s LSD: GH observer (baseline vs discrimination) *p* < 0.0001; SI^P90-104^ observer (baseline vs discrimination) *p* < 0.0001. Download Figure 5-1, TIF file.

### Resocialization fails to rescue emotional discrimination or prosocial deficits induced by adolescent social isolation

**Figure 6.**
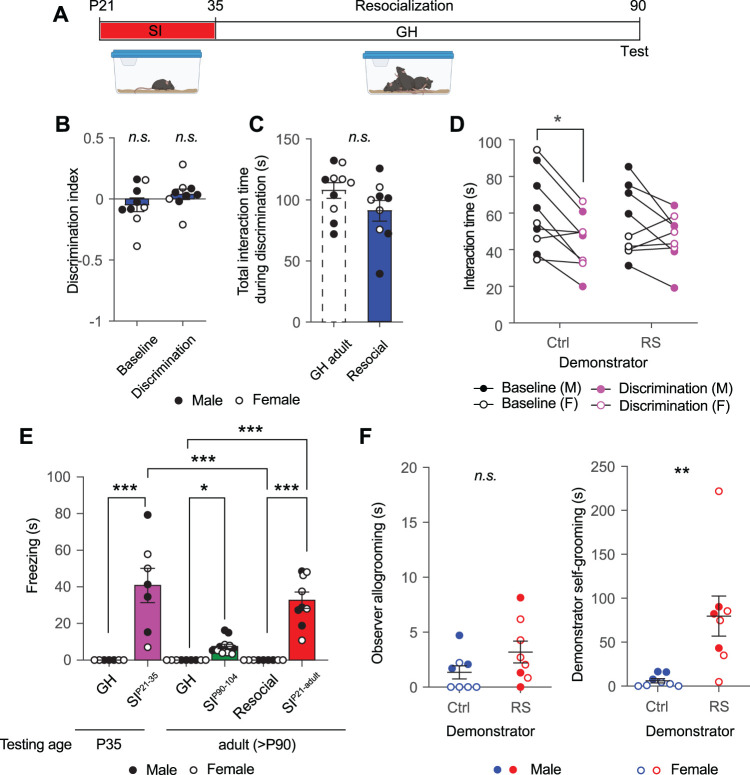
Resocialization of SI^P21–35^ mice from P35 to adult fails to restore emotional discrimination and prosocial allogrooming but reduces freezing during social interaction. ***A***, Timeline of the resocialization experiment. ***B***, DI during baseline and discrimination of resocialized mice. One-sample *t* test compared with 0: baseline *t*_(8)_ = 0.8541, *p* = 0.4179; discrimination *t*_(8)_ = 0.8858, *p* = 0.4016. ***C***, Total interaction time during discrimination is comparable between adult GH and resocialized mice. Mann–Whitney test, *U* = 24, *p* = 0.0947. GH data are the same as in Figure 3*D*. ***D***, Interaction time of resocialized mice with Ctrl versus RS demonstrators during baseline and discrimination. Two-way repeated-measures ANOVA, main effect of demonstrator status: *F*_(1,16)_ = 0.1336, *p* = 0.7195; main effect of experiment phase: *F*_(1,16)_ = 2.990, *p* = 0.1030; interaction between demonstrator status and experiment phase: *F*_(1,16)_ = 1.191, *p* = 0.2912. Uncorrected Fisher's LSD: interaction with Ctrl demonstrator (baseline vs discrimination) *p* = 0.0493; interaction with RS demonstrator (baseline vs discrimination) *p* = 0.3474; baseline interaction (Ctrl vs RS demonstrator) *p* = 0.3182; discrimination phase interaction (Ctrl vs RS demonstrator) *p* = 0.6148. ***E***, Resocialization reduces freezing in all three SI groups. Kruskal–Wallis statistic = 48.76, *p* < 0.0001. Dunn's multiple-comparisons test: GH P35 versus SI^P21–35^ *p* = 0.0004; GH adult versus SI^P21-adult^ *p* < 0.0001; GH adult versus SI^P90–104^ *p* = 0.0440; GH adult versus resocialization *p* > 0.9999; SI^P21-adult^ versus resocialization *p* < 0.0001; SI^P21–35^ versus resocialization *p* = 0.0001. ***F***, Allogrooming of resocialized observers toward Ctrl versus RS demonstrators (left), *W* = 24, *p* = 0.1094; self-grooming of Ctrl versus RS demonstrators in the presence of resocialized observers (right), *W* = 36, *p* = 0.0078; Wilcoxon matched-pairs signed rank test. Extended Data Figure 6-1 is associated with Figure 6.

We next asked whether subsequent social experience could alleviate the social behavior deficits caused by adolescent isolation. SI^P21–35^ mice were resocialized with at least two age- and sex-matched mice for at least 2 months, and their social behaviors were tested in adulthood (Fig. 6*A*). Resocialization failed to restore the ability to discriminate between RS and control demonstrators (Fig. 6*B*,*C*). Like all other groups, resocialized mice spent less time interacting with control demonstrators during discrimination than baseline (Fig. 6*D*). Interestingly, while their interaction time with RS mice was comparable between baseline and discrimination like GH adults (Extended Data Fig. 6-1), the DI was not rescued (phenocopying SI^P21–35^ and SI^P21-adult^; Fig. 6*B* vs Figs. 3*C*, 4*B*). Moreover, SI mice (SI^P21-adult^, SI^P90–104^, and SI^P21–35^) showed a freezing-like behavior during emotional discrimination, i.e., a transient immobility in front of the wired cup containing the demonstrator; this behavior was not observed in GH mice (P35 and adult; Fig. 6*E*; Movies 4, 5). We quantified the total freezing time during the emotional discrimination task (i.e., freezing in front of both cups) and found that resocialization alleviated the freezing phenotype observed in SI^P21–35^ and SI^P21-adult^ mice (Fig. 6*E*). Resocialization also failed to restore prosocial allogrooming toward RS demonstrators (Fig. 6*F*).

10.1523/ENEURO.0441-25.2026.f6-1 Figure 6-1 Behavior of adult GH and resocialized observers during the emotional discrimination test. ***A***, Interaction time with Ctrl demonstrators. Two-way repeated measures ANOVA, main effect of observer housing condition: *F*(1,17) = 0.7385, *p* = 0.4021; main effect of experiment phase: *F*(1,17) = 48.02, *p* < 0.0001; interaction between experiment phase and observer housing condition: *F*(1,17) = 2.013, *p* = 0.1740. Uncorrected Fisher’s LSD: GH observer (baseline vs discrimination) *p* < 0.0001; resocialized observer (baseline vs discrimination) *p* = 0.0014. ***B***, Interaction time with RS demonstrators. Two-way repeated measures ANOVA, main effect of observer housing condition: *F*(1,17) = 6.260, *p* = 0.0229; main effect of experiment phase: *F*(1,17) = 0.1715, *p* = 0.6840; interaction between experiment phase and observer housing condition: *F*(1,17) = 4.056, *p* = 0.0601. Uncorrected Fisher’s LSD: baseline (GH vs resocialization) *p* = 0.3872; discrimination (GH vs resocialization) *p* = 0.0029. Download Figure 6-1, TIF file.

**Movie 4.** An example of GH mouse's behavior during the emotional discrimination test, highlighting its interaction with the demonstrator. [View online]

**Movie 5.** An example of SI mouse's behavior during the emotional discrimination test. Note the freezing behavior when the observer approaches the cup containing the demonstrator. [View online]

## Discussion

Prosocial behavior is broadly defined as any intentional action that benefits another individual (Eisenberg et al., 2007). Such behaviors encompass a wide range of forms and motivations, making them inherently complex to study (Wittek and Bekkers, 2015; Pfattheicher et al., 2022). The Perception–Action Mechanism (PAM) model of empathy (Preston and de Waal, 2002; de Waal and Preston, 2017) provides a useful framework to distinguish between the recognition or perception of another's emotional state (e.g., distress) and the action taken in response (e.g., grooming and comforting). Most rodent work on prosocial behavior has focused on action, but much less is known about discrimination or perception.

In our work, the emotional discrimination assay taps primarily the perception side, i.e., the ability to distinguish a stressed versus unstressed conspecific, while allogrooming toward stressed conspecifics indicates more of the actional side of prosocial behavior. Combining both tests enabled us to dissociate the perceptual and actional components. We found that adolescent social isolation impairs both, whereas adult isolation disrupts the latter but leaves the former intact. Thus, intact emotional state recognition is not sufficient to ensure prosocial helping.

Furthermore, our study revealed a clear temporal sensitivity to social isolation. The inability to rescue the social deficit in SI^P21–35^ mice with resocialization highlights the lasting nature of adolescent social experience in shaping socio-affective capacity. This developmental vulnerability aligns with the concept of a sensitive period, a window during which experience exerts disproportionately strong and lasting effects on brain organization (Knudsen, 2004; Blakemore and Mills, 2014; Nelson et al., 2016; Cheng et al., 2024). A classic work demonstrated that rats isolated between P25 and P45—roughly corresponding to adolescence—exhibited persistent abnormalities in exploratory behavior, which could not be reversed by resocialization (Einon and Morgan, 1976, 1977). Our findings extend this framework to socio-affective processing, indicating that adolescence represents a sensitive period for establishing emotional discrimination abilities (Schneider, 2013).

Why is adolescence uniquely sensitive? One possibility is that critical neural circuits subserving affective recognition and prosocial behavior, such as the medial prefrontal cortex, undergo major developmental reorganization during this period (Caballero et al., 2016; Juraska and Drzewiecki, 2020). Social interaction may help calibrate these circuits to detect and respond to others' affective states. Isolation may lead to maladaptive tuning, reduced synaptic pruning, or altered connectivity. Our data therefore supports a developmental programming view: early social experience sculpts the neural foundations of prosocial/empathic capacities. Furthermore, social play, which is abundant during adolescence but markedly reduced in adulthood, may provide unique experiential input (reciprocity, emotional cue-learning, affiliative touch) that trains recognition/action coupling (Burke et al., 2017; Kellman and Radwan, 2022; Achterberg and Vanderschuren, 2023). Deprivation of peer play may thus remove a key training signal during the sensitive window. In contrast, the persistence of allogrooming deficits after adult isolation suggests that the execution of affiliative helping behavior remains plastic and vulnerable beyond adolescence, even when emotional discrimination is preserved.

Finally, the freezing phenotype provides additional insight into the consequences of social isolation. The transient immobility displayed by isolated mice in front of the demonstrator-containing cup likely reflects social fear and is associated with reduced social investigation (Toth and Neumann, 2013). Its disappearance after resocialization indicates that some isolation-induced abnormalities remain reversible.

In summary, our data identify adolescence as a critical period for the development of prosocial behavior in mice. Brief social isolation during this period suffices to induce persistent deficits in emotional discrimination and affiliative responses, deficits that are not rescued by later social experience. In contrast, adult isolation selectively impairs affiliative helping without abolishing emotional discrimination. These results establish a behavioral framework for investigating how early social experience sculpts neural circuits underlying empathy, prosociality, and stress buffering.

## Synthesis

Reviewing Editor: Sam Golden, The University of Washington Decisions are customarily a result of the Reviewing Editor and the peer reviewers coming together and discussing their recommendations until a consensus is reached. When revisions are invited, a fact-based synthesis statement explaining their decision and outlining what is needed to prepare a revision will be listed below. The following reviewer(s) agreed to reveal their identity: Nancy Padilla-Coreano.

Dear Dr. Zuo,

Your manuscript has been reviewed by two experts in the field. While both reviewers found the manuscript to have strong merit and interest, they have identified several issues that will strengthen the manuscript once addressed. Below, please find their comments, which I hope you will address in a revised submission.

Reviewer 1:

In this manuscript, the authors seek to understand how social isolation in adolescence influences social behavior later in life. In particular, the authors provide insight into how acute and prolonged social isolation during adolescence, in mice, disrupts pro-social behaviors in adulthood. The authors utilize restraint stress in demonstrator conspecifics to promote affiliative pro-social behaviors (sniffing, allogrooming, and self-grooming) in observer subjects. As a measure of emotional discrimination, the authors also utilize a variant of the 3-chamber sociability assay to evaluate the capacity for discrimination of a stressed conspecific (restraint stress) in socially isolated subject mice. The authors provide evidence that early life social isolation disrupts both, the ability to discern the affective state of a stressed conspecific and the propensity to display consolation behavior in the form of allogrooming. Although this work is important for understanding the importance of social experience in adolescence and how it alters social behavior in adulthood, there are some concerns that limit the interpretation and conclusions of the authors.

Major

1. The authors indicate that both sexes were used in this study. In the affiliative experiment, the authors compared allogrooming of the observer mice toward RS demonstrators in male and female animals. Thus, there is likely no sex difference in affiliative pro-social allogrooming in SI observers. However, sex is not considered in the social discrimination experiments. At the very least, the authors must indicate which data points belong to the male animals and which belong to the female animals. This would help with interpretation of the data. For example, Figure 3E - in the GH adult interaction time with RS, 2/5 mice have increase interaction time from BL to discrimination phases, while 3/5 show a decrease, resulting in no discernable difference, on average. There is a similar trend in Figure 3F, Figure 4D, E, and 6D. There is know way of knowing if this is just behavioral variability influencing the statistical outcome or if these differences are due to sex. To statistically compare any sex differences in these experiments the authors will need to increase the sample size.

2. The authors did not test whether resocialization after acute SI (P21-P35) restores affiliative social behavior (allogrooming of stressed conspecific) in adulthood. This is important for the study, as the authors indicate that resocialization does not restore the ability of SI juveniles to recognize the affective state of the demonstrator, but this would be untrue if resocialization restores affiliative behavior.

3. It is interesting to note, and the authors made no mention of it, that there appears to be a difference in the amount of time RS demonstrators spend self-grooming in the presence of a cagemate (Figure 1E right) vs. in the presence of a stranger (Figure 1G right). The authors should test if this is due to sex (like in Figure 1-1D). Also, considering the emotion discrimination test utilized non-cagemates as demonstrators, this could influence the results of these tests. It is possible that the self-grooming of RS demonstrators serves as a cue to observer mice which promotes the ability of the observers to recognize a stressed conspecific. While the control observers were able to recognize the stressed conspecific, it is possible that SI observers require a higher threshold of self-grooming to recognize distress, thus, using cagemate demonstrators, which self-groom more, could promote emotional discrimination. The authors need to perform these tests with cagemates as the demonstrators.

Minor

4. There also appears to be some age dependent differences in the results of the emotional discrimination tests that utilize acute SI. In Figure 4, at the time of testing (P35) the SI animals spend less time interacting with control and RS demonstrators during the discrimination phase relative to BL. These animals are not yet sexually mature and still considered adolescent. However, in Figure 5 and Figure 6, the animals are sexually mature and thus considered young adults. In Figure 5C, the observers do not spend less time interacting with the control demonstrator during discrimination, like they do in adolescence. Also, the SI observers spend more time interacting with the RS demonstrators during the discrimination phase compared to baseline than the P35 animals in Figure 4. This suggests that acutely socially isolated adults are better able to discriminate between a stressed and non-stressed conspecific. However, adults that were acutely socially isolated as juveniles are not able to discriminate a control demonstrator during the discrimination phase (Figure 6D). If Figure 3E left and Figure 6D left are compared this becomes clear. The authors should consider these age-related differences in their experiments and results.

5. Lastly, the authors gave no explanation of why they included the freezing data. There is no mention of its importance in social behavior or what its recovery after resocialization could mean.

Reviewer 2:

This manuscript examines the impact of adolescent social isolation on emotional discrimination and demonstrates that two weeks of isolation during adolescence, but not adulthood, lead to lasting impairments in emotional discrimination. The authors present a well-organized series of experiments that support their main conclusions. However, to further strengthen the manuscript, the authors should address the following point:

Major comments:

1. The manuscript investigates allogrooming behavior toward stressed conspecifics in adulthood following either group housing or prolonged isolation from P21 to adulthood. However, the authors do not address whether a shorter, two-week period of adolescent or adult social isolation impacts prosocial allogrooming. To more precisely determine which aspects of social-emotional behavior are affected by adolescent social isolation, the authors should evaluate allogrooming behavior in prosocial interaction assay following a two-week isolation period. It is currently unclear whether prosocial allogrooming is insufficiently mature at the time of adolescence to be reliably measured, or whether this behaviors simply unaffected by two weeks of isolation.

2. In the current manuscript, it is unclear whether the sensitive window of social isolation specifically affects emotional discrimination and prosocial behavior, or whether these effects reflect broader alterations in general social behavior/sociability. Although the authors primarily discuss findings on social behavior changes following sensitive-window social isolation in rats, they should expand their discussion to incorporate relevant findings from mouse studies as well.

3. The authors highlight that adult social isolation (P90-104) has some impact, particularly an increase in interactions toward stressed mice (Figure 5C), compared with adult GH (Figure 3E). However, the baseline investigation time toward RS appears to differ between Figure 5C and Figure 3E, raising concerns about whether the behavioral effects of adult isolation pointed out by authors are robust. It may strengthen the argument if the authors could statistically compare adult GH mice with P90-104 isolated mice. Alternatively, the authors may consider tempering their conclusions.

Minor comments:

1. Please clarify whether the P21-adult SI mice encounter originally cage-mate mice or novel conspecifics.

## Author Response

Reviewer 1 Major Comments:

1. The authors indicate that both sexes were used in this study. In the affiliative experiment, the authors compared allogrooming of the observer mice toward RS demonstrators in male and female animals. Thus, there is likely no sex difference in affiliative pro-social allogrooming in SI observers. However, sex is not considered in the social discrimination experiments. At the very least, the authors must indicate which data points belong to the male animals and which belong to the female animals. This would help with interpretation of the data. For example, Figure 3E - in the GH adult interaction time with RS, 2/5 mice have increase interaction time from BL to discrimination phases, while 3/5 show a decrease, resulting in no discernable difference, on average. There is a similar trend in Figure 3F, Figure 4D, E, and 6D. There is know way of knowing if this is just behavioral variability influencing the statistical outcome or if these differences are due to sex. To statistically compare any sex differences in these experiments the authors will need to increase the sample size.

We have followed the reviewer's suggestion to indicate the sex of each animal in relevant figures using distinct symbols for males and females. We have also conducted additional experiments to increase the sample size in several groups, including adult GH adult, SIP21-adult, SIP90-104, and resocialization.

GH mice did not exhibit a statistically significant effect of sex on either the discrimination index or the interaction time (Extended Data Fig. 3-1A,B). Among SIP21-adult mice, males and females exhibited comparable discrimination index (Extended Data Fig. 3-1C), but a weak sex difference in interaction time (Extended Data Fig. 3-1D).

With the sex of animal indicated for each data point in the revised figures, our visual inspection found no indication that the mixed increase/decrease patterns as noted by the reviewer would be attributable to the sex of the animals. We acknowledge that these experiments were not originally designed or powered to comprehensively test sex differences across all conditions. Therefore, we interpret these analyses cautiously and present them primarily to improve transparency and aid interpretation of the behavioral data.

2. The authors did not test whether resocialization after acute SI (P21-P35) restores affiliative social behavior (allogrooming of stressed conspecific) in adulthood. This is important for the study, as the authors indicate that resocialization does not restore the ability of SI juveniles to recognize the affective state of the demonstrator, but this would be untrue if resocialization restores affiliative behavior.

We have conducted new experiments to test prosocial allogrooming toward a stressed conspecific in P35 GH and SIP21-35 mice, as well as adult mice that had undergone resocialization following P21-35 SI. Consistent with our observation in the emotional discrimination assay, we found that SIP21-P35 mice did not increase allogrooming toward RS demonstrators, and resocialization failed to rescue this affiliative prosocial behavior. These new data are presented in revised Figures 4F-I and 6F. Thus, our conclusion that resocialization does not rescue SI-induced social deficits is supported not only for emotional discrimination, but also for affiliative prosocial allogrooming.

3. It is interesting to note, and the authors made no mention of it, that there appears to be a difference in the amount of time RS demonstrators spend self-grooming in the presence of a cagemate (Figure 1E right) vs. in the presence of a stranger (Figure 1G right). The authors should test if this is due to sex (like in Figure 1-1D). Also, considering the emotion discrimination test utilized non-cagemates as demonstrators, this could influence the results of these tests. It is possible that the self-grooming of RS demonstrators serves as a cue to observer mice which promotes the ability of the observers to recognize a stressed conspecific.

While the control observers were able to recognize the stressed conspecific, it is possible that SI observers require a higher threshold of self-grooming to recognize distress, thus, using cagemate demonstrators, which self-groom more, could promote emotional discrimination. The authors need to perform these tests with cagemates as the demonstrators.

We appreciate the reviewer's thoughtful comments and have addressed these points with additional analyses and clarification.

Sex-specific analysis of demonstrator self-grooming (cagemate vs stranger).

As suggested by the reviewer, we quantified RS demonstrator self-grooming in the presence of a cagemate versus a stranger (non-cagemate) observer (Fig. 1E vs. 1G, right panels). We found that RS demonstrators self-groomed significantly more in the presence of a cagemate observer than in the presence of a stranger observer (Extended Data Fig. 1-1D). Furthermore, two-way ANOVA revealed a significant main effect of condition (p = 0.0012), but no significant main effect of sex (p = 0.8957), indicating that the difference in demonstrator self-grooming is not explained by sex.

Does demonstrator's self-grooming serve as a cue for observer's allogrooming? To address this possibility, we examined the temporal relationship between demonstrator self- grooming and observer allogrooming. We found that demonstrator self-grooming was not required for initiation of observer allogrooming. Specifically, 14 of 18 observers performed at least one allogrooming episode before the demonstrator exhibited any self-grooming (80 of 246 total allogrooming episodes). This occurred in 4 of 7 observers with cagemate demonstrators (11 of 86 episodes) and 10 of 11 observers with non-cagemate demonstrators (69 of 160 episodes). These results suggest that demonstrator's self-grooming is not a prerequisite for the observer to start allogrooming.

Furthermore, only a relatively small fraction of observer allogrooming occurred during demonstrator self-grooming: 30 of 246 episodes total (12.2%), including 11 of 86 episodes (12.8%) with cagemate demonstrators and 19 of 160 episodes (11.9%) with non-cagemate demonstrators. We also quantified how often observer allogrooming occurred shortly after demonstrator self- grooming onset. Only 28 of 246 allogrooming episodes (11.4%) occurred within 5 seconds, and 47 of 246 episodes (19.1%) within 10 seconds, of demonstrator self-grooming onset. In addition, the average interval between the onset of an allogrooming episode and the onset of the most recent demonstrator self-grooming bout exceeded 1 minute (64.9 s for cagemate demonstrators and 62.6 s for non-cagemate demonstrators).

Finally, despite the difference in RS demonstrator self-grooming between cagemate and non- cagemate conditions (Extended Data Fig. 1-1D), observer allogrooming levels were comparable across these two conditions (Extended Data Fig. 1-1C).

Taken together, these analyses do not support the idea that increased demonstrator self-grooming is a necessary or primary cue driving observer allogrooming or emotional discrimination. Rather, observer prosocial responses can be initiated in the absence of demonstrator self-grooming and are not proportionally increased under conditions in which demonstrator self-grooming is higher.

Feasibility of using cagemate demonstrators in the SI paradigm.

The reviewer raised an interesting hypothesis that "SI observers require a higher threshold of self- grooming to recognize distress, thus, using cagemate demonstrators, which self-groom more, could promote emotional discrimination." However, SI observers are singly housed and therefore do not have cagemates. Implementing a "cagemate demonstrator" emotional discrimination assay in SI animals would require introducing sustained social contact, which would fundamentally interfere with the social isolation manipulation. For this reason, aside from the cagemate condition shown in Fig. 1D,E, our allogrooming and emotional discrimination assays were performed with non- cagemate demonstrators across groups, which also standardizes demonstrator familiarity/novelty between GH and SI observers. We have added statements in the Materials and Methods section clarifying the choice of demonstrators in prosocial interaction assay and emotion discrimination test.

Minor Comments:

4. There also appears to be some age dependent differences in the results of the emotional discrimination tests that utilize acute SI. In Figure 4, at the time of testing (P35) the SI animals spend less time interacting with control and RS demonstrators during the discrimination phase relative to BL. These animals are not yet sexually mature and still considered adolescent. However, in Figure 5 and Figure 6, the animals are sexually mature and thus considered young adults. In Figure 5C, the observers do not spend less time interacting with the control demonstrator during discrimination, like they do in adolescence. Also, the SI observers spend more time interacting with the RS demonstrators during the discrimination phase compared to baseline than the P35 animals in Figure 4. This suggests that acutely socially isolated adults are better able to discriminate between a stressed and non-stressed conspecific. However, adults that were acutely socially isolated as juveniles are not able to discriminate a control demonstrator during the discrimination phase (Figure 6D). If Figure 3E left and Figure 6D left are compared this becomes clear. The authors should consider these age-related differences in their experiments and results.

We thank the reviewer for raising this important point. We tested additional mice in the adult 2- week social isolation (SIP90-104) and the resocialization cohorts, and re-analyzed the emotional discrimination dataset with age taken into consideration. With the expanded dataset, we found:

Adult SI: SIP90-104 observers interacted with control demonstrators less during discrimination than during the baseline phase, just as GH adults (Fig. 5C). However, unlike SIP21-35 and SIP21-adult mice, which show decreased interaction with RS demonstrators during discrimination (Fig. 4E and 3F), SIP90-104 mice show increased interaction with RS demonstrators during discrimination (Fig. 5C). Although this pattern could suggest SIP90-104 mice's stronger differential responses to stressed versus non-stressed conspecifics, direct comparison between adult GH and SIP90-104 mice, as suggested by Reviewer 2 (Comment #3), revealed no significant differences in either interaction time or discrimination index between the two groups (see new Extended Data Fig. 5-1). Together, these data show that the effect of acute SI is age-dependent: 2-week SI in adolescence reduces social engagement towards RS demonstrators, whereas 2-week SI in adulthood does not induce such defects.

Resocialization: As suggested by the reviewer, we compared Figure 3E left panel and Figure 6D left panel directly (see new Extended Data Fig. 6-1). We found that the interaction time of resocialized observers with control demonstrators followed the same pattern as adult GH observers (Extended Data Fig. 6-1A). Interestingly, while resocialized observers spent comparable time interacting with RS demonstrators during baseline vs discrimination (like what we observed with adult GH observers), their interaction time with RS demonstrators was significantly shorter than that of GH observers during discrimination but not baseline (Extended Fig. 6-1B). Furthermore, the discrimination index was still not rescued (phenocopying SIP21-35 and SIP21-adult; Fig. 6B vs. Fig.

3C and 4B). Together with the abolishment of the freezing behavior during emotional discrimination (Fig. 6E, also see answers to the next question) and the lack of rescue of the prosocial behavior (new Fig. 6F), our findings suggest that while resocialization can rescue some of the social deficits, it is insufficient to fully restore emotional discrimination and the associated prosocial behavior.

5. Lastly, the authors gave no explanation of why they included the freezing data. There is no mention of its importance in social behavior or what its recovery after resocialization could mean.

The "freezing" behavior here refers to the transient immobility of the observer in front of the wired cup containing the demonstrator during the emotional discrimination test. This behavior was exhibited exclusively by SI animals (SIP21-adult, SIP90-104, and SIP21-35) but was not observed in GH mice (P35 and adult; compare Movie 4 and 5). Resocialized mice no longer exhibited freezing, suggesting that this aspect of isolation-induced behavioral abnormality is reversible, even when emotional discrimination and allogrooming were not fully restored.

The freezing behavior we observed likely reflects a social fear of the animals and may be associated with reduced social investigation. We therefore reported freezing as an additional readout to document a robust SI phenotype present during the discrimination assay, and to ensure that the social interaction metrics are interpreted in the context of a potential defensive response.

Reviewer 2 Major comments:

1. The manuscript investigates allogrooming behavior toward stressed conspecifics in adulthood following either group housing or prolonged isolation from P21 to adulthood. However, the authors do not address whether a shorter, two-week period of adolescent or adult social isolation impacts prosocial allogrooming. To more precisely determine which aspects of social-emotional behavior are affected by adolescent social isolation, the authors should evaluate allogrooming behavior in prosocial interaction assay following a two-week isolation period. It is currently unclear whether prosocial allogrooming is insufficiently mature at the time of adolescence to be reliably measured, or whether this behaviors simply unaffected by two weeks of isolation.

2. In the current manuscript, it is unclear whether the sensitive window of social isolation specifically affects emotional discrimination and prosocial behavior, or whether these effects reflect broader alterations in general social behavior/sociability. Although the authors primarily discuss findings on social behavior changes following sensitive-window social isolation in rats, they should expand their discussion to incorporate relevant findings from mouse studies as well.

We have added the prosocial allogrooming test for acute adolescent isolation (SIP21-35) and adult isolation (SIP90-104) mice. We found that SIP21-P35 mice showed an allogrooming deficit toward RS demonstrators (Fig. 4F and G). 2-month resocialization failed to rescue this (Fig. 6F; also see Response to Reviewer 1, Comment #2).

To our surprise, SIP90-104 mice also exhibited an allogrooming deficit toward RS demonstrators (new Fig. 5F), despite their intact ability to distinguish RS from control mice (Fig. 5A). These new data suggest that prosocial behavior involves at least two components: (1) the ability to identify a conspecific's emotional state (assessed by emotional discrimination, impaired only by adolescent SI), and (2) the decision/execution of affiliative helping behavior (which can be disrupted by SI across ages). Intact emotional state recognition is not sufficient for prosocial helping (in the case of SIP90-104). Adolescent isolation (SIP21-35) engenders a persistent deficit in emotional state recognition, consistent with an adolescent sensitive period for social behavior development.

We thank the reviewer for encouraging these additional experiments, which significantly improved this manuscript. We have incorporated these new data together with additional discussion in the revised manuscript as suggested by the reviewer.

3. The authors highlight that adult social isolation (P90-104) has some impact, particularly an increase in interactions toward stressed mice (Figure 5C), compared with adult GH (Figure 3E). However, the baseline investigation time toward RS appears to differ between Figure 5C and Figure 3E, raising concerns about whether the behavioral effects of adult isolation pointed out by authors are robust. It may strengthen the argument if the authors could statistically compare adult GH mice with P90-104 isolated mice. Alternatively, the authors may consider tempering their conclusions.

We thank the reviewer for this helpful suggestion. We collected more data and performed additional analyses to compare adult GH and SIP90-104 mice in emotional discrimination (Figure 5- 1). We found no significant difference between GH adult and SIP90-104 mice in interaction time during either the baseline or discrimination phase, and no significant difference in discrimination index between the two groups. Thus, we have tempered our conclusion accordingly in the revised manuscript.

Minor comments:

1. Please clarify whether the P21-adult SI mice encounter originally cage-mate mice or novel conspecifics.

The SIP21-adult mice encountered novel conspecifics in the allogrooming assay. We have clarified this in the revised Materials and Methods section.
