# Supplementary figures and images for: Adolescent Social Isolation Induces Persistent Impairments in Emotional Discrimination and Helping Behavior
Source: eNeuro. 2026 Jul 7;13(7):ENEURO.0441-25.2026. doi: 10.1523/ENEURO.0441-25.2026 (PMC13362189; doi:10.1523/ENEURO.0441-25.2026)

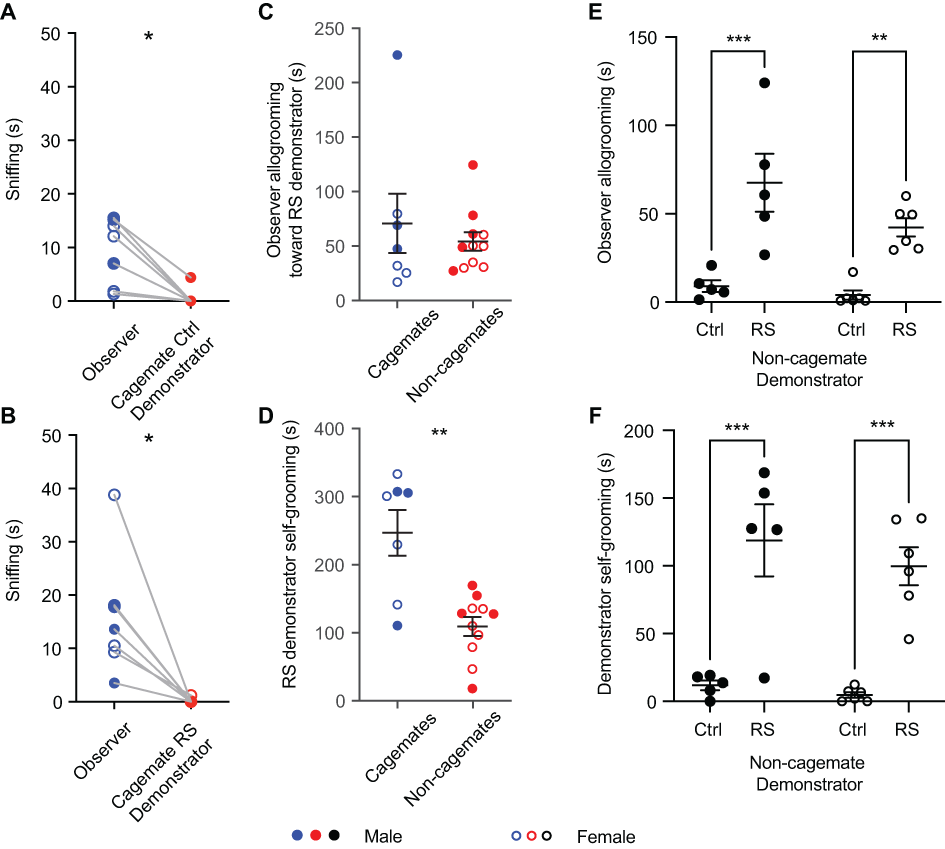

Supplement: Figure 1-1 — Behavioral analyses of observers and demonstrators during social interaction under different conditions. A, Observer mice show significantly more sniffing toward cagemate Ctrl demonstrators than vice versa. Wilcoxon matched-pairs signed rank test, W = -28, p = 0.0156. B, Observer mice show significantly more sniffing toward cagemate RS demonstrators than vice versa. Wilcoxon matched-pairs signed rank test, W = -28, p = 0.0156. C, Observer mice exhibit comparable amount of allogrooming toward cagemate vs non-cagemate RS demonstrators. Mann-Whitney test: U = 37, p = 0.9298. D, Cagemate RS demonstrators show more self-grooming than non-cagemate RS demonstrators during the prosocial test. Mann-Whitney test, U = 8, p = 0.0041. E, Allogrooming of male and female observers toward non-cage demonstrators. Two-way repeated measures ANOVA, main effect of demonstrator status: F(1,9) = 35.16, p = 0.0002; main effect of sex: F(1,9) = 3.411, p = 0.0978; interaction between demonstrator status and sex: F(1,9) = 1.532, p = 0.2471. Uncorrected Fisher’s LSD: male (Ctrl vs RS demonstrators) p = 0.0009; female (Ctrl vs RS demonstrator) p = 0.0069. F, Self-grooming of male and female demonstrators in present of non-cage observers. Two-way repeated measures ANOVA, main effect of demonstrator status: F(1,9) = 59.99, p < 0.0001; main effect of sex: F(1,9) = 0.6936, p = 0.4265; interaction between demonstrator status and sex: F(1,9) = 0.2199, p = 0.6555. Uncorrected Fisher’s LSD: male (Ctrl vs RS demonstrators) p = 0.0004; female (Ctrl vs RS demonstrators) p = 0.0004. Download Figure 1-1, TIF file. [file eneuro-13-ENEURO.0441-25.2026-s007.tif]

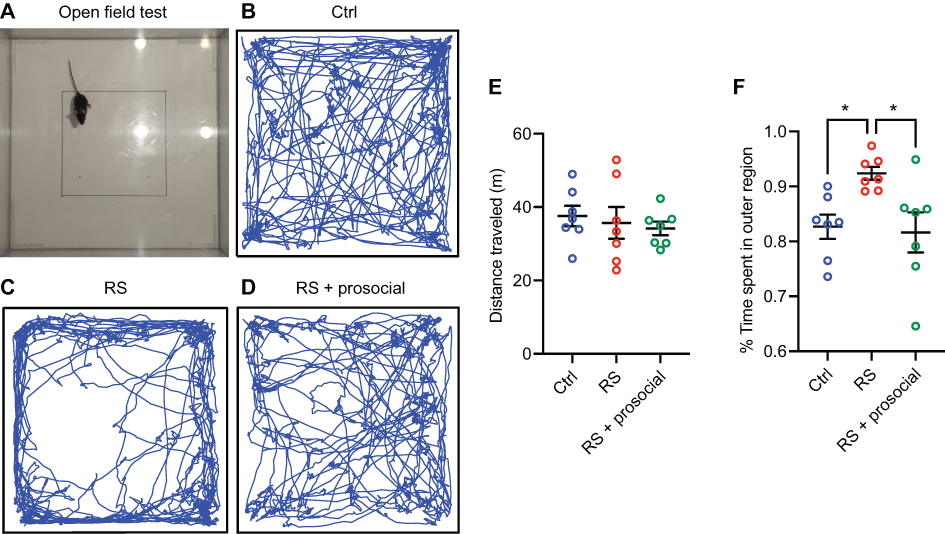

Supplement: Figure 1-2 — Allogrooming reduces the anxiety of RS demonstrators as assessed by the open field test. A, Schematic of the open field test. B, Movement trajectory of a control mouse over 15 min. C, Movement trajectory of a mouse recently subjected to 30 min RS following by 15 min single-housing recovery. D, Movement trajectory of a mouse recently subjected to 30 min RS followed by 15 min of prosocial interaction. E, Total distance traveled for all the experimental groups. One-way ANOVA, F(2, 18) = 0.2904, p = 0.7514. F, Fraction of time spent in the peripheral region of the open field for all experimental groups. One-way ANOVA, F(2, 18) = 5.360, p = 0.0149; Tukey’s multiple comparisons test: Ctrl vs RS p = 0.0387; Ctrl vs RS + prosocial p = 0.9554; RS vs RS + prosocial p = 0.0214. n = 7 female mice. Download Figure 1-2, TIF file. [file eneuro-13-ENEURO.0441-25.2026-s008.tif]

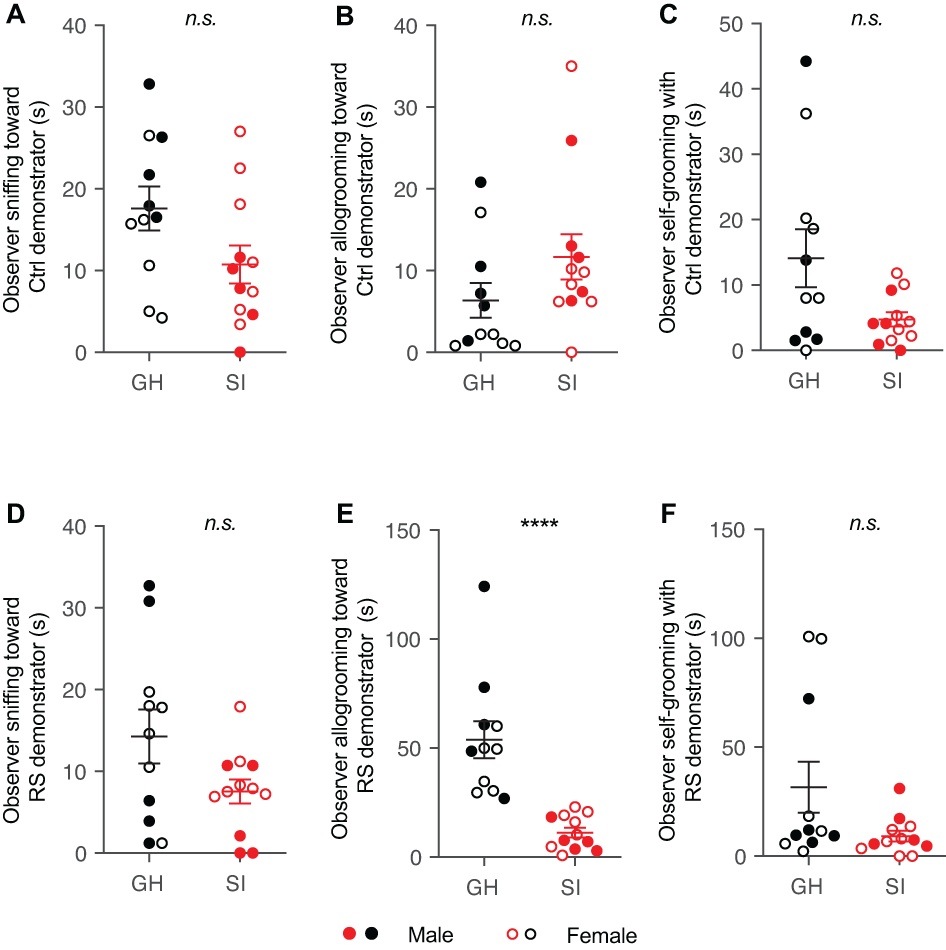

Supplement: Figure 2-1 — Behavioral comparison between adult GH and SIP21-adult observers toward Ctrl vs RS demonstrators. A, Adult GH and SIP21-adult mice show comparable sniffing toward Ctrl demonstrators. U = 40, p = 0.1179. B, Adult GH and SIP21-adult mice show comparable allogrooming toward Ctrl demonstrators. U = 39, p = 0.1002. C, Adult GH and SIP21-adult mice show comparable self-grooming during interaction with Ctrl demonstrators. U = 44, p = 0.1839. D, Adult GH and SIP21-adult mice show comparable sniffing toward RS demonstrators. U = 44, p = 0.1841. E, Adult GH mice exhibit significantly more allogrooming than SIP21-adult mice toward RS demonstrators. U = 0, p < 0.0001. F, Adult GH and SIP21-adult mice show comparable self-grooming during interaction with RS demonstrators. U = 41, p = 0.1301. Mann-Whitney test for all comparisons. Download Figure 2-1, TIF file. [file eneuro-13-ENEURO.0441-25.2026-s009.tif]

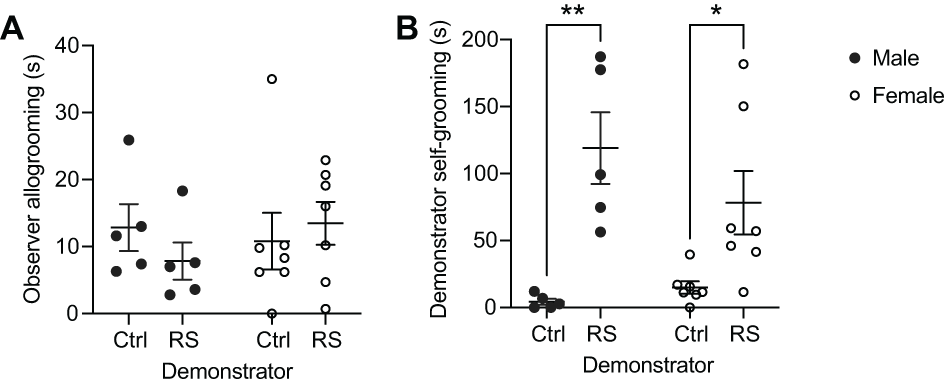

Supplement: Figure 2-2 — Both male and female SIP21-adult observers show impaired allogrooming despite enhanced self-grooming of RS demonstrators. A, Allogrooming of male and female SIP21-adult observers toward Ctrl and RS demonstrators. Two-way repeated measures ANOVA, main effect of demonstrator status: F(1,10) = 0.07436, p = 0.7906; main effect of sex: F(1,10) = 0.3592, p = 0.5623; interaction between demonstrator status and sex: F(1,10) = 0.8038, p = 0.3910. B, Self-grooming of male and female demonstrators in the presence of SIP21-adult observers. Two-way repeated measures ANOVA, main effect of demonstrator status: F(1,10) = 22.18, p = 0.0008; main effect of sex: F(1,10) = 0.7383, p = 0.4103; interaction between demonstrator status and sex: F(1,10) = 1.850, p = 0.2037. Uncorrected Fisher’s LSD: male (Ctrl vs RS demonstrators) p = 0.0026; female (Ctrl vs RS demonstrators) p = 0.0267. Download Figure 2-2, TIF file. [file eneuro-13-ENEURO.0441-25.2026-s010.tif]

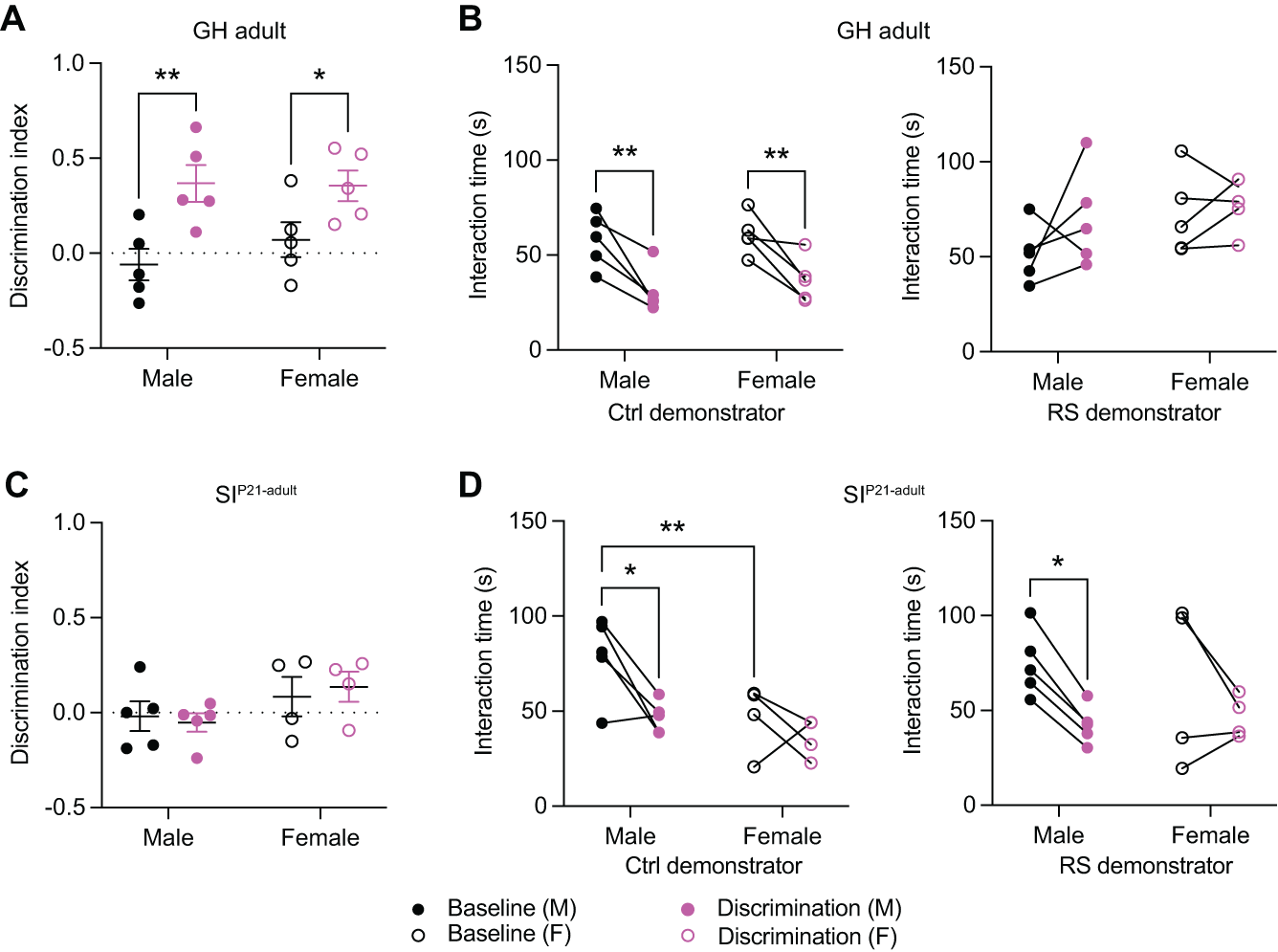

Supplement: Figure 3-1 — Behavior of GH adults and SIP21-adult observers during the emotional discrimination test. A, Discrimination index of male and female adult GH observers. Two-way repeated measures ANOVA, main effect of sex: F(1,8) = 0.3170, p = 0.5889; main effect of experiment phase: F(1,8) = 27.58, p = 0.0008; interaction between sex and experiment phase: F(1,8) = 1.121, p = 0.3207. Uncorrected Fisher’s LSD: male (baseline vs discrimination) p = 0.0021; female (baseline vs discrimination) p = 0.0180. B, Interaction time of male and female adult GH observers. Ctrl demonstrator: Two-way repeated measures ANOVA, main effect of sex: F(1,8) = 0.4931, p = 0.5025; main effect of experiment phase: F(1,8) = 33.06, p = 0.0004; interaction between sex and experiment phase: F(1,8) = 0.1101, p = 0.7485. Uncorrected Fisher’s LSD: male (baseline vs discrimination) p = 0.0026; female (baseline vs discrimination) p = 0.0050. RS demonstrator: Two-way repeated measures ANOVA, main effect of sex: F(1,8) = 2.351, p = 0.1637; main effect of experiment phase: F(1,8) = 2.030, p = 0.1920; interaction between sex and experiment phase: F(1,8) = 0.6178, p = 0.4545. C, Discrimination index of male and female SIP21-adult observers. Two-way repeated measures ANOVA, main effect of sex: F(1,7) = 2.829, p = 0.1365; main effect of experiment phase: F(1,7) = 0.02022, p = 0.8909; interaction between sex and experiment phase: F(1,7) = 0.3944, p = 0.5499. D, Interaction time of male and female SIP21-adult observers. Ctrl demonstrator: Two-way repeated measures ANOVA, main effect of sex: F(1,7) = 9.211, p = 0.0190; main effect of experiment phase: F(1,7) = 7.942, p = 0.0258; interaction between sex and experiment phase: F(1,7) = 1.943, p = 0.2060. Uncorrected Fisher’s LSD: male (baseline vs discrimination) p = 0.0160; female (baseline vs discrimination) p = 0.3711; baseline (male vs female) p = 0.0080; discrimination (male vs female) p = 0.3127. RS demonstrator: Two-way repeated measures ANOVA, main effect of sex: F(1,7) [file eneuro-13-ENEURO.0441-25.2026-s011.tif]

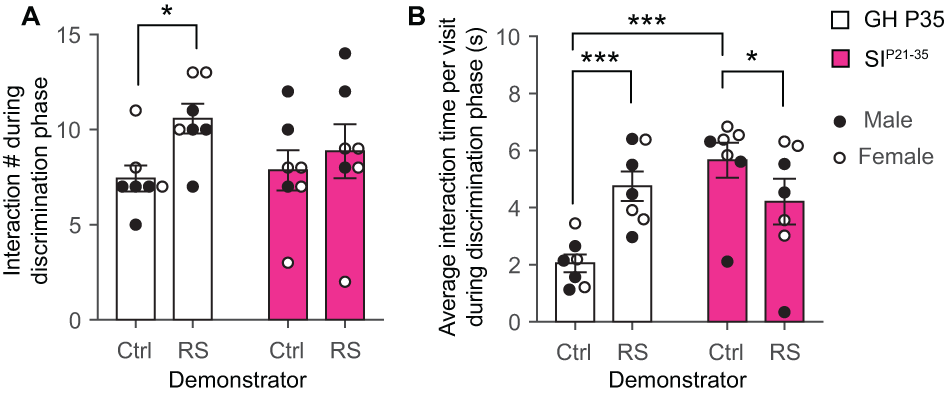

Supplement: Figure 4-1 — Behavior of P35 GH and SIP21-35 observers during the discrimination phase of the emotional discrimination test. A, Total interaction number of P35 GH and SIP21-35 mice toward Ctrl vs RS demonstrators during discrimination. Two-way repeated measures ANOVA, main effect of demonstrator status: F(1,12) = 7.763, p = 0.0165; main effect of observer housing condition: F(1,12) = 0.2656, p = 0.6157; interaction between demonstrator status and observer housing condition: F(1,12) = 2.077, p = 0.1751. Uncorrected Fisher’s LSD: GH observer interaction (Ctrl vs RS demonstrators) p = 0.0113; SIP21-35 observer interaction (Ctrl vs RS demonstrators) p = 0.3603. B, Average interaction time per visit of P35 GH and SIP21-35 mice toward Ctrl vs RS demonstrators during discrimination. Two-way repeated measures ANOVA, main effect of demonstrator status: F(1,12) = 2.951, p = 0.1115; main effect of observer housing condition: F(1,12) = 4.234, p = 0.0620; interaction between demonstrator status and observer housing condition: F(1,12) = 32.82, p < 0.0001. Uncorrected Fisher’s LSD: interaction with Ctrl demonstrator (GH vs SIP21-35 observers) p = 0.0002; interaction with RS demonstrator (GH vs SIP21-35 observers) p = 0.5231; GH observer interaction (Ctrl vs RS demonstrators) p = 0.0002; SIP21-35 observer interaction (Ctrl vs RS demonstrators) p = 0.015. Download Figure 4-1, TIF file. [file eneuro-13-ENEURO.0441-25.2026-s012.tif]

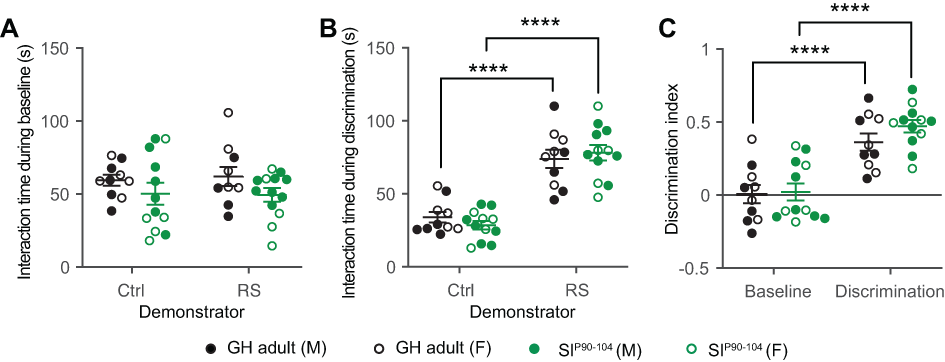

Supplement: Figure 5-1 — Behavior of adult GH and SIP90-104 observers during the emotional discrimination test. A, Interaction time during baseline. Two-way repeated measures ANOVA, main effect of demonstrator status: F(1,20) = 0.03242, p = 0.8589; main effect of observer housing condition: F(1,20) = 2.586, p = 0.1235; interaction between demonstrator status and observer housing condition: F(1,20) = 0.1132, p = 0.7400. B, Interaction time during discrimination. Two-way repeated measures ANOVA, main effect of demonstrator status: F(1,20) = 97.77, p < 0.0001; main effect of observer housing condition: F(1,20) = 0.01804, p = 0.8945; interaction between demonstrator status and observer housing condition: F(1,20) = 1.139, p = 0.2985. Uncorrected Fisher’s LSD: GH observer (Ctrl vs RS demonstrators) p < 0.0001; SIP90-104 observer (Ctrl vs RS demonstrators) p < 0.0001. C, Discrimination index. Two-way repeated measures ANOVA, main effect of experiment phase: F(1,20) = 74.09, p < 0.0001; main effect of observer housing condition: F(1,20) = 0.9367, p = 0.3447; interaction between experiment phase and observer housing condition: F(1,20) = 1.005, p = 0.3280. Uncorrected Fisher’s LSD: GH observer (baseline vs discrimination) p < 0.0001; SIP90-104 observer (baseline vs discrimination) p < 0.0001. Download Figure 5-1, TIF file. [file eneuro-13-ENEURO.0441-25.2026-s013.tif]

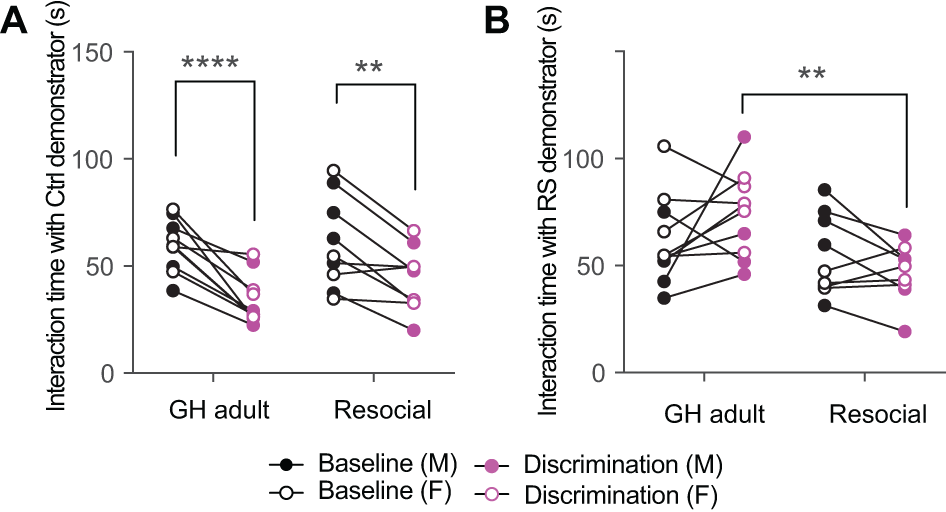

Supplement: Figure 6-1 — Behavior of adult GH and resocialized observers during the emotional discrimination test. A, Interaction time with Ctrl demonstrators. Two-way repeated measures ANOVA, main effect of observer housing condition: F(1,17) = 0.7385, p = 0.4021; main effect of experiment phase: F(1,17) = 48.02, p < 0.0001; interaction between experiment phase and observer housing condition: F(1,17) = 2.013, p = 0.1740. Uncorrected Fisher’s LSD: GH observer (baseline vs discrimination) p < 0.0001; resocialized observer (baseline vs discrimination) p = 0.0014. B, Interaction time with RS demonstrators. Two-way repeated measures ANOVA, main effect of observer housing condition: F(1,17) = 6.260, p = 0.0229; main effect of experiment phase: F(1,17) = 0.1715, p = 0.6840; interaction between experiment phase and observer housing condition: F(1,17) = 4.056, p = 0.0601. Uncorrected Fisher’s LSD: baseline (GH vs resocialization) p = 0.3872; discrimination (GH vs resocialization) p = 0.0029. Download Figure 6-1, TIF file. [file eneuro-13-ENEURO.0441-25.2026-s014.tif]
